# Genomic Aberrations in Lung Adenocarcinoma in Never Smokers

**DOI:** 10.1371/journal.pone.0015145

**Published:** 2010-12-06

**Authors:** Bastien Job, Alain Bernheim, Michèle Beau-Faller, Sophie Camilleri-Broët, Philippe Girard, Paul Hofman, Julien Mazières, Saloua Toujani, Ludovic Lacroix, Julien Laffaire, Philippe Dessen, Pierre Fouret, for the LG Investigators

**Affiliations:** 1 Plate-forme de Biologie intégrée, Institut de recherche intégrée en Cancérologie à Villejuif, Villejuif, France; 2 INSERM Génétique des tumeurs U985, INSERM, Villejuif, France; 3 Laboratoire de Biochimie et de Biologie Moléculaire (Hôpital de Hautepierre), CHU Strasbourg, Strasbourg, France; 4 INSERM JE2492, INSERM, Kremlin-Bicêtre, France; 5 Université Paris-Sud, Kremlin-Bicêtre, France; 6 Département Thoracique, Institut Mutualiste Montsouris, Paris, France; 7 Laboratoire de Pathologie Clinique et Expérimentale (Hôpital Pasteur), CHU Nice, Nice, France; 8 Unité de Cancérologie Cervico Thoracique (Hôpital Larrey), CHU Toulouse, Toulouse, France; 9 Laboratoire de Recherche Translationnelle, Institut de cancérologie Gustave-Roussy, Villejuif, France; 10 Ligue Nationale contre le Cancer, Paris, France; 11 Université Pierre et Marie Curie, Paris, France; National Cancer Institute, United States of America

## Abstract

**Background:**

Lung cancer in never smokers would rank as the seventh most common cause of cancer death worldwide.

**Methods and Findings:**

We performed high-resolution array comparative genomic hybridization analysis of lung adenocarcinoma in sixty never smokers and identified fourteen new minimal common regions (MCR) of gain or loss, of which five contained a single gene (*MOCS2*, *NSUN3*, *KHDRBS2*, *SNTG1* and *ST18*). One larger MCR of gain contained NSD1. One focal amplification and nine gains contained *FUS*. *NSD1* and *FUS* are oncogenes hitherto not known to be associated with lung cancer. FISH showed that the amplicon containing *FUS* was joined to the next telomeric amplicon at 16p11.2. *FUS* was over-expressed in 10 tumors with gain of 16p11.2 compared to 30 tumors without that gain. Other cancer genes present in aberrations included *ARNT*, *BCL9*, *CDK4*, *CDKN2B*, *EGFR*, *ERBB2*, *MDM2*, *MDM4*, *MET*, *MYC* and *KRAS*. Unsupervised hierarchical clustering with adjustment for false-discovery rate revealed clusters differing by the level and pattern of aberrations and displaying particular tumor characteristics. One cluster was strongly associated with gain of *MYC*. Another cluster was characterized by extensive losses containing tumor suppressor genes of which *RB1* and *WRN*. Tumors in that cluster frequently harbored a central scar-like fibrosis. A third cluster was associated with gains on 7p and 7q, containing *ETV1* and *BRAF*, and displayed the highest rate of EGFR mutations. SNP array analysis validated copy-number aberrations and revealed that *RB1* and *WRN* were altered by recurrent copy-neutral loss of heterozygosity.

**Conclusions:**

The present study has uncovered new aberrations containing cancer genes. The oncogene *FUS* is a candidate gene in the 16p region that is frequently gained in never smokers. Multiple genetic pathways defined by gains of *MYC*, deletions of *RB1* and *WRN* or gains on 7p and 7q are involved in lung adenocarcinoma in never smokers.

## Introduction

Tobacco smoking is the main avoidable cause of lung cancer. However, lung cancer also occurs in never smokers and would rank as the seventh most common cause of cancer death worldwide [1], [2]. In France, lung cancer in never smokers accounted in the year 2000 for 17% and 4% of lung cancer deaths among women and men, respectively [3].

Lung cancer in never smokers occurs more frequently among women, and it favors the adenocarcinoma histological type [4]. One of the most striking distinctions is the observed differential response to drugs that target the epidermal growth factor receptor (EGFR). Compared with smokers, never smokers treated with these agents have higher response rates to treatment [5], [6].

*EGFR* mutations in lung cancer are more frequent in never smokers and are exclusive with *KRAS* mutations [7], [8], [9], [10], [11]. Mutations in *HER2* also target never smokers [12]. The transversion/transition ratio and the distribution of *TP53* and *KRAS* mutations differ according to smoking status [13], [14], [15], [16]. The complex mutational signatures of lung cancer cells in smokers reflect the cocktail of carcinogens in tobacco smoke and their proclivities for particular bases [17].

While it is well established that specific DNA sequence abnormalities are linked to smoking status, other oncogenomic events are less well known among never smokers. In most genomic studies, the proportion of never smokers is unknown or small compared to that of smokers. Few separate studies of aberrations in never smokers have been performed, mainly in patients from East Asia [18], [19]. Allelic imbalances were infrequent in never smokers with lung adenocarcinoma [20], although in Chinese never smokers their pattern appeared distinct [18]. In Chinese never smokers the most frequent aberration was gain of 16p [19]. In the largest study of the lung adenocarcinoma genome, never smoker status was associated, although not significantly, with amplification of 7p-q and 16p and deletion of 10q and 15q [21]. Preliminary studies also indicate a relationship between smoking history and *EML4-ALK* fusions [22].

The catalogue of copy-number aberrations may lead to the identification of imbalances encompassing genes that contribute to the development or progression of lung cancer [23]. Here, we tried to accrue knowledge of aberrations occurring in lung adenocarcinoma in never smokers with the goal to uncover new aberrations that would include cancer genes.

## Materials and Methods

Detailed methods on inclusion of patients, processing of samples, *EGFR* and *KRAS* sequencing, oligonucleotide aCGH analysis, genomic PCR, fluorescent in situ hybridization studies, gene expression analysis and SNP array analysis are available in supplementary information (Material and Methods S1).

### Patients and samples

The project, referred as the Lung Genes (LG) study, involved 13 centers in France. The 60 patients were never smokers - defined following current consensus guidelines [24], [25] as persons with a lifetime exposure of less than 100 cigarettes. All patients had been treated by surgery. The pathological diagnosis was reviewed and cases for which a doubt about the primary site in the lung remained were excluded.

The research has been approved by the Institut National du Cancer review board as part of the Programme National d'Excellence Spécialisé Poumon. Writen consent has been obtained from study patients for the use of their lung samples.

Genomic DNA and RNA were extracted from frozen tumor sections and the HCC827 cell line, obtained from ATCC. The cell line was authenticated by comparison of its Agilent aCGH profile with the previously published whole genome tiling path aCGH profile [26].

### Sequencing of *EGFR* and *KRAS*

*EGFR* exons 18, 19, 20, 21 and *KRAS* exons 2 and 3 were directly sequenced in both sense and antisense directions from at least two independent amplifications.

### Oligonucleotide aCGH analysis

Genomic DNA was analyzed using 244K Whole Human Genome (G4411B) microarrays (Agilent Technologies, Santa Clara, CA, USA). The data are described in accordance with MIAME guidelines and have been deposited in ArrayExpress (http://www.ebi.ac.uk/arrayexpress) under E-TABM-926 accession number.

The threshold for gain and loss was abs(log2ratio)>0.25 for a minimum of 5 consecutive probes. Focal amplifications were considered for aberrations showing a log2(ratio) >1.58 and extending less than 5 Mb. Minimal common regions (MCR) were identified with STAC v1.2 [27] and by using both the frequency-confidence and footprint methods at lower and higher stringencies (confidence >0.95 and >0.995, respectively). MCR were manually reviewed to validate breakpoints and to discard copy-number variants. For hierarchical clustering, Euclidean distances and Ward's construction method were used. The bootstrap tests were performed using the R environment package Pvclust [28]. Cluster-associated aberrations were identified using ANOVA with P values adjusted for their false-discovery rate using the Benjamini-Hochberg method [29] The P values (F-test) for the association of clusters with clinicopathological variables were adjusted for multiple testing using Bonferroni correction.

### Genomic PCR

Quantification of *FUS* genomic DNA was performed in Taqman® assays (Applera, Villebon-sur-Yvette, France) using primers and probes that were designed using Primer3 software.

### Fluorescence *in situ* hybridization (FISH) studies

FISH was performed on tumor touch-imprinted slides.

### Gene expression analysis

The gene expression analysis encompassed HG-U1133 plus 2.0 Affymetrix array data in a subset of 40 samples belonging to an ongoing study (not published). Expression of probe sets in the 16p11.2 region was compared with the t-test.

Quantification of *FUS* mRNA expression was performed in pre-designed Taqman®gene expression assays.

### SNP array analysis

SNP array genotyping was carried out using the Illumina “HumanCNV370-Quad” array (Illumina, Inc., San Diego, CA) in the subset of 40 samples belonging to an ongoing study (not published). Individual cases with aCGH profiles delineating an aberration were selected for cross-validation by SNP array profiles. The aCGH profile in the region of aberration was compared to the corresponding SNP array profile for each selected case using the Integrated Genome Browser (http://www.bioviz.org/igb/).

For assessment of copy-neutral loss of heterozygosity (LOH), only segments with at least 10 consecutive SNPs showing a LOH and a copy number equal to 2 were considered.

## Results

### Clinicopathological characteristics

The clinicopathological characteristics are shown in Table S1. The median age was 69 years (interquartile range, 59 to 77). Patients were more frequently women (88%). Pathological stages were stage I in 32 cases (53%), stage II in 6 cases (10%), stage III in 21 cases (35%), and stage IV in 1 case (2%). The median tumor size was 31 mm (interquartile range, 25 to 40). The tumors were well-differentiated in 34 cases (57%), moderately differentiated in 7 cases (12%) and poorly differentiated in 19 cases (32%). They comprised a broncholioalveolar component in 28 cases (47%). Central fibrosis was present in 19 cases (32%). Tumor expressed the NKX-2 protein in 57 cases (95%).

### Genome complexity

The percentages of aberrant genome (AG) were calculated for each case (mean 17%, median 16%, range 0 to 64%). The percentages of gains (mean 9%, median 7%, range 0% to 31%) and of losses (mean 8%, median 6%, range 0% to 41%) were similar and correlated (R2 = 0.102, P = 0.01). Those percentages were not correlated when cases with low levels of AG (<5%) belonging mainly to cluster A1 (see below) were excluded (R2 = 0.002, P = 0.84) (Figure S1).

### Partition of tumors into clusters

**Figure 1:**
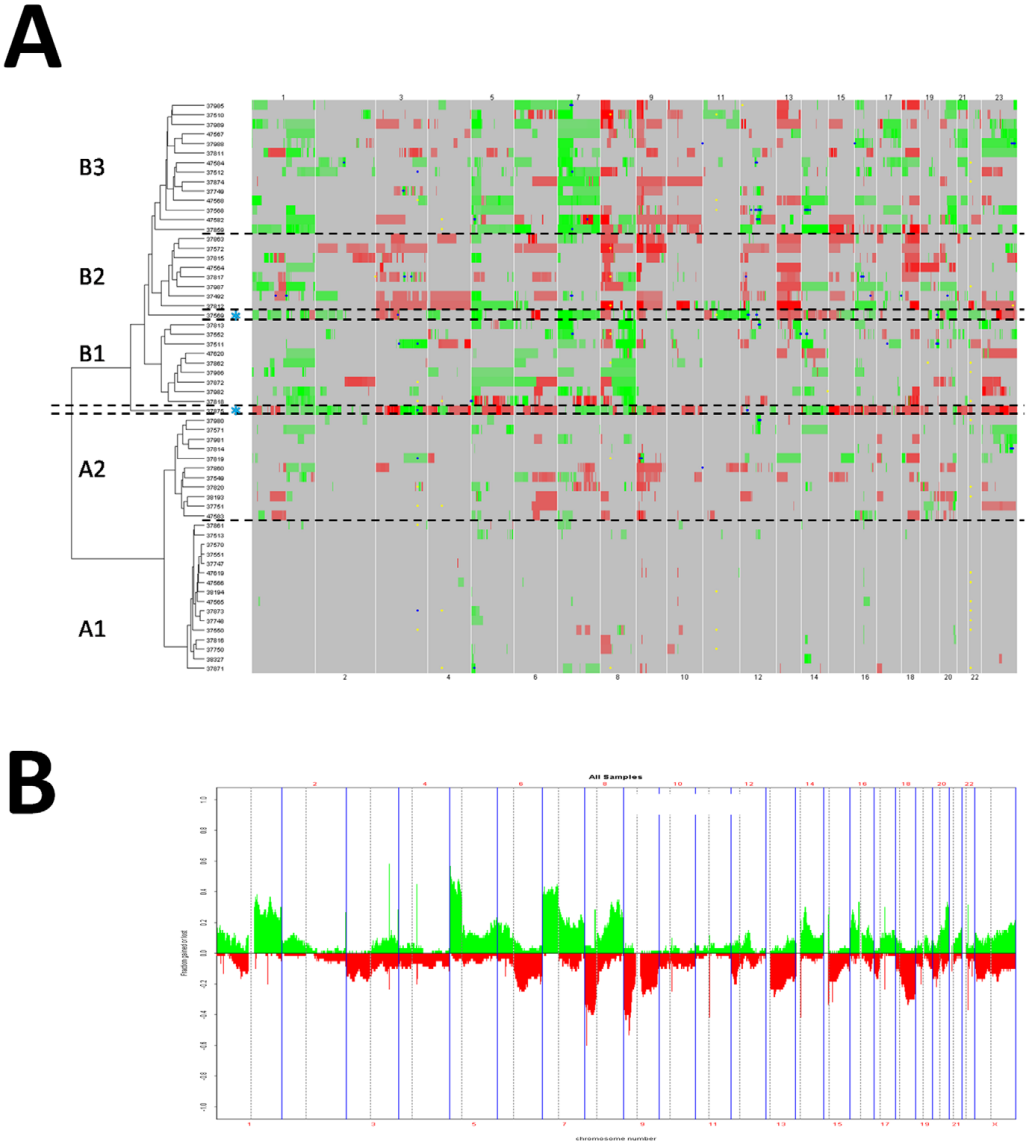
Aberrations using aCGH analysis in 60 never smokers with lung adenocarcinoma. **Panel A.** Heat map of gains (green color) and losses (red color) by chromosome generated by non supervised hierarchical clustering. Small blue or yellow dot indicate gains with log2(ratio)>1.5 and losses with log2(ratio)<−1.5, respectively. Blue star (*): two outliers (37875 between classes A and B and 37569 between clusters B1 and B2). **Panel B.** Distribution of gains (green color) and losses (red color) along the genome.

A non supervised hierarchical clustering analysis revealed two main classes A and B, which could be further subdivided into 2 clusters A1 (n = 16) and A2 (n = 11) for A and into 3 clusters B1 (n = 9), B2 (n = 9) and B3 (n = 14) for B (Figure 1). An assessment of the uncertainty in hierarchical clustering is provided in Figure S2.

Clusters differed by their AG percentages (P<0.001; Figure S3) and their aberration patterns. Cluster A1 was characterized by few aberrations, which comprised recurring gains on 5p, 7p, 14q and 20q, and losses on 8p (Table S2). In cluster A2 the level of AG (mean 12%, range 2 to 18%) was higher than in cluster A1 (mean 2%, range 0 to 4%). The aberration pattern in cluster A2 was different from the patterns of clusters B1, B2 and B3, indicating that cluster A2 was not a cluster belonging to class B with reduced amplitude in the aberrations. Cluster A2 had more losses (9%) than gains (7%), while cluster B1 had twice more gains (13%) than losses (6%). Notably, cluster B1 was characterized by the occurrence in every case of a gain on 8q. Cluster B2 was characterized by more losses (21%) than gains (10%) with a distinctive combination of numerous and frequent losses on 3p, 8p and 13. Cluster B3 was defined by gains on 7p and 7q, together with gains on 17q, 21, and less frequently X. One outlier between class A and class B was characterized by a uniquely high level of AG (64%), which was distributed in both gains (23%) and losses (41%); another outlier between cluster B1 and B2 displayed a gain of the whole chromosome 12.

By ANOVA, gains including oncogenes and losses including tumor suppressor genes were significantly associated after adjustment for their false discovery rate with particular clusters (Table S3). *MYC* at 8q24.21 was gained in 100% of cases in cluster B1 (adjusted P = 6.00E-05). *BRAF* was included in a region extending 1.27 Mb at 7q34 that was gained in 64% of cases in cluster B3 (adjusted P = 0.001). Other gains on 7q including *ELN*, *HIP1*, *CREB3L2* and *KIAA1549* were associated with cluster B3. The gains on 7p containing *CARD11*, *ETV1* and *IKZF1* were observed in 78% to 92% of cases of cluster B3. Several regions on 13q that included *CDX2*, *BRCA2*, *RB1* and *ERCC5* were lost in 77% to 88% of cases in cluster B2. *WRN* at 8p12 was the single gene present in a deleted region in 88% of cases in cluster B2 (adjusted P = 0.002).

The five clusters differed by their association with a central scar-like fibrosis (P = 0.03 after Bonferroni correction), which was more frequent in cluster B2 (7/9 cases) compared to other clusters (12/50 cases). They did not differ with regard to other clinicopathological characteristics.

### Relationships of clusters with abnormalities in *EGFR* and *KRAS*

Forty tumors (67%) harbored *EGFR* mutations (Table S4). The four *KRAS* mutations were observed in four *EGFR* wild-type cases.

The prevalence of *EGFR* mutations differed with clusters (P = 0.004), gains on 7p (P = 0.04) and AG percentages (P<0.001). *EGFR* mutations remained associated with clusters after adjustment for AG percentages and gain on 7p (P = 0.05). Cluster B3 was characterized by the highest frequency of gains on 7p (93%), and the highest frequency of *EGFR* mutations (93%), although these abnormalities did not coincide. Most gains on 7p (80%) and every case with an amplification spanning *EGFR* were associated with *EGFR* mutation. Nineteen *EGFR* mutations were seen in cases with no gain on 7p.

While every gain on 7p included *EGFR*, only 5 of 14 gains on 12p included *KRAS* either wild-type (3 cases) or mutated (2 cases). The distribution of mutations or gains involving *EGFR* or *KRAS* is displayed in Figure S4. The 10 cases without abnormality involving *EGFR* or *KRAS* belonged to clusters A1 (9 cases) or A2 (1 case with 2% AG). Amplifications of *MET* and *ERBB2* occurred with a gain on 7p and an *EGFR* mutation, respectively.

### Distribution of recurrent aberrations

Recurrent gains were observed on 1q, 5p, 7p, 8q and 16p in >20% of cases and on 5q, 6p, 7q, 14 q, 16q, 17q, 20q, 21q and Xq in 10% to 20% of cases (Figure 1). Recurrent losses were observed on 8p, 9p, 9q, 13q and 18q in >20% of cases and on 3p, 6q, 12p, 15q, 17p, 18p, 20p, Xp and Xq in 10% to 20% of cases. The proportion of tumors harboring gains on 5p or 7p and losses on 8p or 9p exceeded 40%. A comparison with previously reported large aberrations is shown in Table S5.

### Minimal common regions

MCRs of gain were identified on 1q, 2p, 5p, 5q, 7p, 7q, 8q, 12p, 12q, 14q, 18p and 20q (Table 1). Their mean width was 879 Kb (range 109 to 2927). The maximum log2(ratio) ranged from 0.53 to 3.13. The twenty-two MCRs contained 152 coding genes, including *BCL9*, *ARNT*, *MDM4*, *NSD1*, *EGFR*, *MYC* and *MDM2*, as well as 6 *miRNA*. The highest frequency of recurring gains (62%) was noted at 5p13.33 that contained *TERT* and *CLPTM1L*. The MCR containing *EGFR* was involved in 43% of cases. A 171 Kb MCR at 20q13.33 contained only *mir-646*. Nine MCR contained between 1 and 5 coding genes, five MCR between 7 and 9 coding genes, and four MCR more than 10 coding genes. The MCR of gains were compared to previously published regions of gain in four representative studies [21], [30], [31], [32]. As shown in Table 1, out of eight MCR that did not overlap with previously reported gains, one MCR contained a single gene (*MCOS2*) and two MCR contained only three genes.

**Table 1:** Minimal common regions of gain in lung adenocarcinoma in 60 never smokers.

Cytoband	Start*	End	Width (Kb)	Coding genes (n)	Log2 ratio§	Frequency	Coding genes&	miRNA gene	Previously reported overlapping region$
1q21.1	144967396	146188634	1221	9	2.46	33%	**BCL9** CHD1L PRKAB2 FMO5 GJA5 GJA8 ACP6 GPR89B PDZK1P1	-	Kim et al.
1q21.2	148910280	149167813	257	4	1.07	37%	**ARNT** CTSK CTSS HORMAD1	-	Kim et al.; Weir et al.
1q21.3	152773939	153124479	350	4	1.07	38%	ADAR CHRNB2 KCNN3 UBE2Q1	-	Kim et al. Tonon et al.
1q32.1	202347395	203140588	793	9	1.02	37%	KISS1 REN ETNK2 GOLT1A **MDM4** PIK3C2B LRRN2 PLEKHA6 PPP1R15B	-	Weir et al.
2p21	43387367	46315293	2927	15	0.97	13%	PPM1B SIX3 SLC3A1 PREPL LRPPRC SIX2 DYNC2LI1 SRBD1 ABCG5 ABCG8 C2orf34 PLEKHH2 UNQ6975 hCG_1645220 PRKCE	-	Weir et al.
5p15.33	1280621	1654137	373	5	1.74	62%	TERT SLC6A3 CLPTM1L LPCAT1 SDHAP3	-	Weir et al.
5p15.32	4965919	5075638	109	0	1.58	50%	-	-	
5p12-5p11	45366792	46008894	642	0	1.09	37%	-	-	
5q11.2	52373558	52735879	362	1	0.53	18%	MOCS2	-	
5q35.2-5q35.3	176142617	178360147	2217	41	0.59	22%	UNC5A HK3 UIMC1 ZNF346 FGFR4 **NSD1** RAB24 PRELID1 MXD3 LMAN2 RGS14 SLC34A1 PFN3 F12 GRK6 PRR7 DBN1 PDLIM7 DOK3 DDX41 FLJ10404 TMED9 B4GALT7 FAM153A PROP1 FAM153C RPL19P9 N4BP3 RMND5B NHP2 GMCL1L HNRNPAB AGXT2L2 COL23A1 CLK4 ZNF354A ZNF354B ZFP2 FLJ31183 ZNF454 GRM6	-	
5q35.3	179513778	180194523	680	7	0.59	23%	FLT4 MAPK9 GFPT2 CNOT6 SCGB3A1 OR2Y1 MGAT1	-	
7p11.2	54766919	56108248	1341	12	3.13	42%	**EGFR** SEC61G LANCL2 ECOP PSPHL SEPT14 FKBP9L CCT6A GBAS PSPH MRPS17 ZNF713	-	Kim et al.; Tonon et al.; Weir et al.; Zhao et al.
7q11.2	64860558	65616940	756	7	0.6	28%	DKFZp434F142 CCT6P1 ASL GUSB RCP9 VKORC1L1 TPST1	-	Zhao et al.
8q24.21	128611272	129784296	1173	3	1.3	35%	**MYC** PVT1 TMEM75	mir-1205 mir-1206 mir-1207 mir-1208	Kim et al.; Tonon et al.; Weir et al.; Zhao et al.
8q24.23	139213139	139712071	498	0	1.17	28%	-	-	
12p11.21	31151912	31558797	406	3	0.81	12%	FAM60A FLJ13224 OVOS2	-	
12q15	67079640	68051228	971	9	2.41	15%	**MDM2** RAP1B NUP107 hCG_1757335 CPM SLC35E3 MGC5370 CPSF6 LYZ	mir-1279	Tonon et al.; Weir et al.; Zhao et al.
14q13.2-14q21.1	35346936	38036164	2689	15	2.58	30%	MBIP NKX2-1 NKX2-8 PAX9 MIPOL1 FOXA1 BRMS1L STELLAR SFTA3 FLJ42220 TTC6 C14orf25 SSTR1 CLEC14A SLC25A21	-	Tonon et al.; Weir et al.; Zhao et al.
18p11.32	634728	831011	196	3	1.05	12%	TYMS ENOSF1 C18orf56	-	Kim et al.; Tonon et al.
20q13.2	51212268	51703066	490	2	0.81	30%	ZNF217 RP4-724E16.2	-	Zhao et al.
20q13.31	54675484	55408554	733	3	0.82	33%	BMP7 SPO11 RAE1	-	
20q13.33	58147620	58319505	171	0	0.81	32%	-	mir-646	Tonon et al.

*hg 18 assembly;

§ Maximum value;

& Oncogenes according to the 2010 april Cancer Gene Census^38^ are in bold characters; genes for which a somatic mutation has been reported in lung cancer (Cosmic database april 2010) are underlined;

$ Weir et al.^21^; Tonon et al.^28^; Kim et al.^29^; Zhao et al.^30^

MCRs of loss were identified on 1p, 3q, 6q, 8q, 9p, 16q and 20p (Table 2). Their mean width was 560 Kb (range 20 to 1703). The minimum log2(ratio) ranged from −0.43 to −1.19. In four cases it was <−1. The nine MCRs contained 18 coding genes, including *CDKN2B* for which the highest frequency of losses (53%) was noted. Five MCRs contained only one coding gene, and three MCRs between 3 and 6 coding genes. As shown in Table 2, six MCR of loss did not overlap with previously reported losses. Four of these MCR contained a single gene (*NSUN3*, *KHDRBS2*, *SNTG1* and *ST18*) and one MCR contained four genes.

**Table 2:** Minimal common region of loss in lung adenocarcinoma in 60 never smokers.

Cytoband	Start*	End	Width (Kb)	Genes (n)	Log2 ratio§	Frequency	Coding genes&	miRNA genes	Previously reported overlapping region$
1p22.1	92942530	93381319	439	3	−0.43	18%	EVI5 MTF2 FAM69A	-	Tonon et al.
3q11.2	95327585	95416019	88	1	−0.74	15%	NSUN3	-	
6q11.1	62484326	62985285	501	1	−0.8	12%	KHDRBS2	-	
8q11.22	50928921	51329519	401	1	−1.19	20%	SNTG1	-	
8q11.23	53446319	53494813	48	1	−1.16	15%	ST18	-	
9p21.3	21998396	22018473	20	1	−1.18	53%	**CDKN2B**	-	Tonon et al.; Weir et al.; Zhao et al.
9q21.13	74982746	75362791	380	0	−0.56	28%	-	-	
16q23.1	76281180	77681228	1400	4	−0.43	10%	WWOX VAT1L NUDT7 CLEC3A	-	
20p12.1	13515731	15279357	1763	6	−1.05	22%	FLRT3 ESF1 TASP1 C20orf7 SEL1L2 MACROD2	-	Weir et al.

*hg 18 assembly;

§ Minimum value;

& Tumor suppressor genes according to the 2010 april Cancer Gene Census^38^ are in bold characters;

$ Weir et al.^21^; Tonon et al.^30^; Kim et al.^31^; Zhao et al.^32^

### Top focal amplifications

Twenty-seven focal (extending less than 5 Mb) amplifications were observed on 2q, 3p, 3q, 5p, 7p, 7q, 9p, 12p, 12q, 14q, 16p, 17q, 20q and Xq (Table 3). Their mean width was 643 Kb (range 14 to 4567 Kb). The maximum log2(ratio) ranged from 1.61 to 4.37. The 27 focal amplifications contained 114 coding genes, including *MET*, *KRAS*, *CDK4*, *FUS* and *ERBB2*, as well as two isolated *miRNA* and a cluster of 14 *miRNA*. Twenty-four amplifications were observed once. The three amplifications containing *TRIO*, *DKFZp564N2472* and *CDK4* were observed twice. Nine amplifications contained between 1 and 3 coding genes, ten between 4 and 6 coding genes, and three >10 coding genes. Eleven amplifications overlapped with previously reported gains.

**Table 3:** Top focal (<5Mb) amplifications (log2 ratio >1.58) not contributing to minimal common regions of gain.

Slide	Cytoband	Start*	End	Width (Kb)	Coding genes (n)	Log2 ratio§	Coding genes&	miRNA	Previously reported overlapping region$
47584	2q14.2	120120731	120483864	363	2	1.69	PTPN4 TMEM177	-	Tonon et al.
37511	3p11.2	87661697	88571168	909	4	1.98	HTR1F CGGBP1 ZNF654 C3orf38	-	
37749	3q13.12	109377362	109440377	63	0	1.68	-	-	
37749	3q13.13	109999496	110354933	355	4	1.63	GUCA1C MORC1 TRAT1 FLJ22763	-	
37749	3q13.2	113240470	113522551	282	4	1.64	TMPRSS7 C3orf52 GCET2 SLC9A10	-	
37817	3q13.31	115227473	115456050	229	3	2.63	DRD3 ZNF80 QTRTD1	-	
37817	3q22.3	138959945	139026309	66	1	2.88	SOX14	-	Tonon et al.
37871, 47582	5p15.2	14167833	14668598	501	3	1.71	TRIO UNQ1870 FAM105A	-	Kim et al.
37492, 37985	7p12.1	52760845	53085803	325	1	3.21	DKFZp564N2472	-	
47582	7q31.2	115355561	116206401	851	6	2.13	**MET** # CAV1 CAV2 TFEC TES tcag7.929	-	Tonon et al.; Weir et al.; Zhao et al.
37819	9p22.3	15490609	15525984	35	0	1.8	-	-	
37819	9p21.3	21418395	21862731	444	3	1.67	IFNA1 IFNE1 MTAP	mir-31	
37875	12p12.1	24937842	25295816	358	5	4.37	**KRAS** BCAT1 LRMP CASC1 LYRM5	-	Tonon et al.; Weir et al.
37569	12p12.1-12p11.21	26213455	30754588	4541	19	4.32	SSPN MED21 FGFR1OP2 TM7SF3 C12orf11 C12orf71 STK38L ARNTL2 PTHLH PPFIBP1 KLHDC5 MRPS35 REP15 CCDC91 ERGIC2 FAR2 TMTC1 OVCH1 IPO8	-	Tonon et al.
37568	12q13.13-12q13.2	53027533	53669280	642	12	2.23	ITGA5 ZNF385A GPR84 NCKAP1L PDE1B PPP1R1A LACRT DCD GTSF1 GLYCAM1 KIAA0748 MUCL1	-	
37569, 47584	12q14.1	56426027	56440483	14	3	3.18	**CDK4** TSPAN31 MARCH9	-	Weir et al.; Zhao et al.
37568	14q22.1	51202459	51815854	613	4	2.78	NID2 C14orf166 GNG2 PTGDR	-	
37817	16p12.1	24688436	24743419	55	0	2.64	-	-	Tonon et al.
37817	16p12.1	24882738	25043384	161	0	2.69	-	-	Tonon et al.
37817	16p12.1	25462373	25608504	146	0	2.59	-	-	Tonon et al.
37817	16p11.2	30013241	30048741	36	3	2.56	MAPK3 GDPD3 YPEL3	-	
37817	16p11.2	30715645	30901000	185	4	2.47	CTF1 BCL7C FBXL19 ORAI3 (NCRNA00095)	-	
37817	16p11.2	31097645	31215554	118	4	2.59	**FUS** PYCARD PYDC1 TRIM72	-	
37511	17q12	35096506	35253366	157	5	3.76	**ERBB2** GRB7 IKZF3 C17orf37 PERLD1	-	Kim et al.; Weir et al.; Zhao et al.
37492	20q11.21	31371257	31524747	153	2	1.61	SNTA1 CDK5RAP1	-	
37814	Xq27.1	138207177	139111284	904	4	1.64	F9 MCF2 ATP11C RP11-35F15.2	mir-505	
37988	Xq27.3	144986917	149553420	4567	18	1.78	FMR1 FTHL8 FMR1NB ASFMR1 AFF2 IDS MAGEA9 MAGEA11 HSFX1 TMEM185A CXorf40A MAGEA9B HSFX2 MAGEA8 CXorf40B FLJ16423 FLJ44451 MAMLD1	mir-513c mir-513b mir-513a-1 mir-513a-2 mir-506 mir-507 mir-508 mir-509-2 mir-509-3 mir-509-1 mir-510 mir-514-1 mir-514-2 mir-514-3	

*hg 18 assembly;

§ Maximum value;

& Oncogenes according to the 2010 april Cancer Gene Census^38^ are in bold characters; genes for which a somatic mutation has been reported in lung cancer (Cosmic database april 2010) are underlined;

# MET except 20 Kb at the 3′ part of the gene;

$ Weir et al.^21^; Tonon et al.^30^; Kim et al.^31^; Zhao et al.^32^

### Copy-neutral loss of heterozygosity

Forty-five of regions of interest which had been identified by aCGH (Tables 1, 2 and 3) could be evaluated by SNP analysis in 40 tumors. Thirty-nine regions were cross-validated by the SNP array profiles. An example is shown in Figure S5.

The SNP arrays could be analyzed for detection of copy-neutral LOH in 23 cases. The 17 remaining samples were not informative for LOH. Two-hundred and five regions displayed recurring copy-neutral LOH. MCR of recurring copy-neutral LOH with a frequency >20% are shown in Table S6. Among tumor suppressor genes that were present in losses identified by aCGH, *RB1* and *WRN* were also present within copy-neutral LOH MCRs.

### The 16p11.2 region harboring the oncogene *FUS*

**Figure 2:**
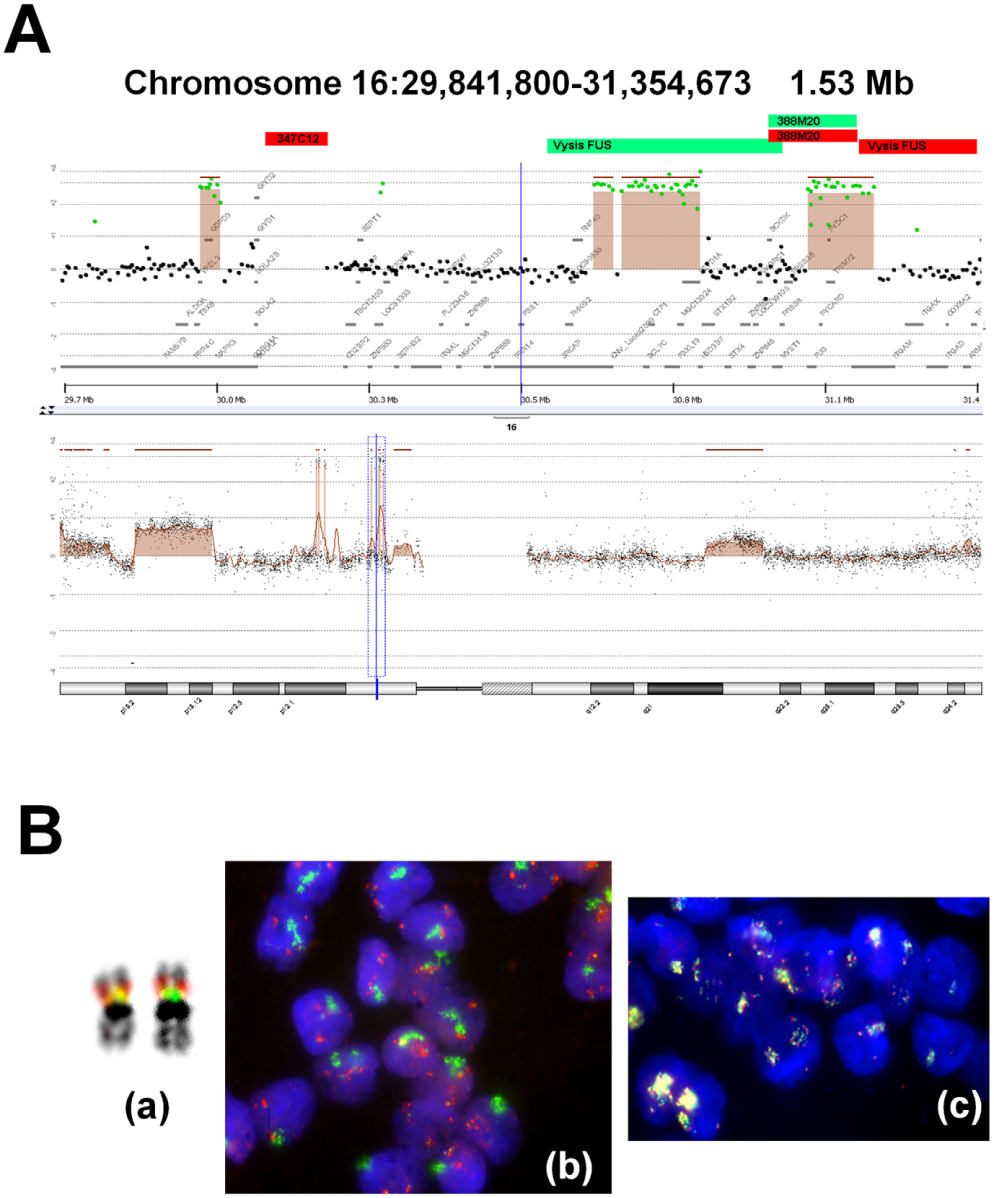
Amplicons on 16p11.2 in case 37817 using aCGH and FISH analyses. **Panel A.** aCGH analysis. Below: chromosome 16 diagram; the blue line limits the 16p11.2 region represented above. Above: aCGH profile for the enlarged 16p11.2 region showing the complex amplification. The dots are individual oligonucleotides that are in green when they are gained; a brown color, enhanced by an horizontal line, show the region of copy-number alteration segmented by the algorithm. The p telomere is to the left, the centromere to the right. The location and color of probes used for FISH are indicated as red or green squares at the upper part of the aCGH profile. **Panel B.** Examples of FISH results for the 16p11.2 region. (a) Normal chromosome 16 from a normal blood mitosis, in DAPI inversed colors showing the specific heterochromatin secondary constriction of the long arm. Although separated by less than a 1 Mb, RP11-347C12 (red) is slightly more telomeric than RP11-388M20 (green), although they are fused for a large part. (b) The same probes on case 37817 cells showing a distinct pattern of amplification. (c) Combination of Vysis FUS probes with RP11-388M20 (red) that show a co-localization of the three probes on the amplicon even in decondensed HS.

The short arm of chromosome 16 displayed high-level focal amplifications in case 37817. There were two distinct regions of amplification that were separated by >4 Mb and extended 0.92 Mb and 1.20 Mb at 16p12.1 and at 16p11.2, respectively (Table 3). Each region comprised three peaks, which extended 36 Kb to 185 Kb and were spaced by 140 to 670 Kb. The 16p11.2 amplicons shown in Figure 2 harbored *FUS*, 12 other coding genes, and one long non-coding RNA gene. Nine additional cases demonstrated gains of a smaller amplitude encompassing *FUS*.

Real-time quantitative PCR assays in case 37817 showed a strong increase (>30 times) in *FUS* copy number compared to *AQP8* and *AMPD2*, which were located in copy-neutral regions.

The 16p11.2 region was explored by FISH by using two BAC clones (RP11-388M20 and RP11-347C12). The former completely covered *FUS,* while the latter was 745 Kb telomeric to it in the region <30,109–30,290Mb> (Figure 2). Both BAC were co-hybridized on normal metaphases and nuclei, and the signals were superposed. When co-hybridized on tumor cells from case 37817, two independent gene amplification homogeneously staining region (HSR) patterns appeared (Figure 2), demonstrating that the breakpoint of an unknown chromosomal translocation separated the two amplified segments (the telomeric amplification revealed by RP11-347C12 was not apparent in the aGGH results as this region was not covered by Agilent oligoprobes). Then, the amplicon containing *FUS* was characterized using RP11-388M20 together with the Vysis break apart probe. The BAC probe was stained in the same color as the centromeric part of the Vysis probe, but in a color different from that of the telomeric part. The probes were found amplified with a HSR pattern and co-localized in tumor cells, delimiting the previous breakpoint from 30,27 to 30,50 Mb. Furthermore, the co-localization suggested that the two amplicons <30,71–30,90Mb> and <31,09–31,21> were physically linked, as the 0,2Mb region <30,90–31,09Mb> was not amplified.

As shown in Figure S6, analysis of gene expression array data showed that four probe sets (1565717_s_at, 200959_at, 215744_at and 217370_x_at) interrogating *FUS* were significantly overexpressed in the subgroup of 10 tumors harboring a 16p gain compared with 30 tumors without such gain.

Real-time PCR gene expression assay established that *FUS* mRNA relative levels were 4 times higher in tumor 37817 (mean ΔCT 2.6) compared to NCI-HCC827 cell line (mean ΔCT 4.6), which displayed no gain on 16p.

## Discussion

We used a high-resolution aCGH to analyze aberrations that occurred in lung adenocarcinoma in 60 never smokers. We identified new MCR of gain or loss and new amplifications. Furthermore, unsupervised hierarchical clustering showed that tumors could be classified into clusters exhibiting different levels and pattern of aberrations, which contained cancer genes. Clusters differed by their tumor characteristics.

Fourteen MCR of gain (eight regions) or loss (six regions) did not overlap with regions that were previously reported in four representative studies [21], [30], [31], [32]. Out of our newly described MCR, five contained a single coding gene (*MCOS2*, *NSUN3*, *KHDRBS2*, *SNTG1* and *ST18*) and may be considered as high-priority regions for further studies. Somatic mutations in genes within narrow MCR, including *FLT4*, *MAPK9*, *SPO11 and KHDRBS2*, have been reported in cancers (COSMIC v48 release). Among single genes encompassed by MCR of loss, *ST18* was present in a 48 Kb MCR. *ST18* was found lost, hypermethylated and its mRNA downregulated in breast cancer [33].

Some newly uncovered aberrations contained oncogenes such as *FUS* at 16p11.2 and *NSD1* at 5q35.2–q35.3, whose association with lung cancer has hitherto not been reported. A gain on 16p has been previously associated with lung cancer in never smokers, although the association was not significant after multiple testing [19], [21]. We note that the association with never smoker status may be confounded by ethnicity or sex [34]. We found that the oncogene *FUS* was present in a high-level narrow amplification at 16p11.2 in one tumor (37818). It should be noted that nine other tumors displayed gains encompassing *FUS*, although the gene was first identified from a single patient. Furthermore, in the gene expression analysis the mean FUS expression level was compared between the 10 tumors displaying the 16p gain and 30 tumors without such gain. As *FUS* was found overexpressed in the subgroup with 16p gain, it was identified as a candidate gene from 10 tumors. Originally described as the result of translocations in myxoid liposarcoma [35], *FUS* encodes a TET protein that exerts roles in transcription and splicing and functions in several aspects of growth control and DNA repair [36]. Here, the aberration in tumor 37818 consisted of three closely spaced amplicons, suggesting amplification through breakage-fusion-bridge cycles [37]. Furthermore, FISH showed that the amplicon containing *FUS* was joined with the next telomeric amplicon in a HSR. The whole 16p11.2 region appeared highly rearranged as shown by the lack of FISH co-localization of the BAC covering *FUS* with a farther telomeric BAC. Among genes present in the 16p11.2 amplicon only *FUS* has until now been reported as altered by somatic simple mutation in cancer (Cosmic v48 release). While our data are consistent with *FUS* as a candidate gene in lung adenocarcinoma in never smokers, they do not prove that *FUS* is the functional target of the amplification. It is essential to systematically analyze using functional assays the whole 16p11.2 region.

To pinpoint cancer genes, we used a census that is conducted with relatively conservative criteria [38]. It is remarkable that we found many cancer genes that were previously reported in aberrations in lung cancer, including *BCL9*, *ARNT*, *MDM4*, *EGFR*, *MYC*, *MDM2*, *CDKN2B*, *MET*, *CDK4*, and *ERBB2*. Large aberrations are also consistent with the literature [19], [21], [23], [26], [31]. The gain containing *TERT* was reported as the most frequent event (78%) in early lung cancer [39]. *TERT* was included in this study within a MCR of gains with a high frequency (62%). At 5p15.2 *TRIO* was previously identified in a focal amplification and was found differentially expressed in early-stage lung cancer [40]. At 5p13 *GOLPH3* was recently established as a new oncogene that was gained in lung and other cancers [41]. It was frequently gained in our study without being included in a MCR or a focal amplification. At 14q13.2–14q21.1 we found a MCR of gain containing *MBIP*, *NKX2-1*, *NKX2-8* and *PAX9*, whose cooperation is involved in lung tumorigenesis [42]. Overlapping with previously reported regions, other MCR were often delineated with better precision. We identified a 390Kb MCR at 20q13.2, reported by Zhao et al. [32], that contained two genes of which *ZNF217* was found mutated in lung cancer. Another MCR at 20q13.33, reported by Tonon et al. [30], contained only *mir-646*.

We used hierarchical clustering to determine whether tumors were heterogeneous and whether there were cluster-specific aberrations, which could have been hidden in the study of the whole cohort. Tumors could be classified into five clusters that differed by their AG percentages and aberration patterns. Interestingly, the compendium of cancer genes that were present in cluster-associated recurring aberrations was to a large extent different from the list of MCR-associated cancer genes except for *MYC*. Present in a MCR in the whole cohort, *MYC* was also strongly associated with one cluster (cluster B1), where it was gained in every case. As point mutations in *MYC* do not occur in lung cancer, the gain of *MYC* could be important for lung cancer classification in never smokers. The tumor suppressor gene *WRN*, which encodes a helicase, was the single gene present in a narrow region at 8p12 that was frequently lost in cluster B2. *WRN* has been reported to undergo epigenetic inactivation through CpG island promoter hypermethylation in about one-third of non-small cell lung cancer [43]. Other losses associated with cluster B2 were located on 13q and included *RB1*, which is frequently altered in lung cancer [16], and three other tumor suppressor genes. Another gain that was associated with cluster B3 included *BRAF*, whose mutation has been reported in 3% of non-small cell lung cancer [44]. There were other noteworthy gains on 7p and 7q, however, among which that of *ETV1* was the most strongly associated with cluster B3. The results presented here support heterogeneity in the genetic pathways in lung adenocarcinoma in never smokers. This view is strengthened by the association of cluster B2 with scar-like tumor fibrosis, a desmoplastic reaction which is common in localized peripheral lung adenocarcinoma, and of cluster B3 with the highest rate of *EGFR* mutation (93%) as well as the highest rate of the co-occurrence of *EFGR* mutations and gains or amplifications on 7p (86%).

*EGFR* mutations were found in 68% of cases in our study, a high rate similar to those reported in never or former light smokers in two recent studies [45], [46], while mutations in *KRAS* were infrequent. *EGFR* mutations were exclusive of *KRAS* mutations, a consistent observation suggesting that *EGFR* and *KRAS* mutations signal through a common pathway. The fact that every gain on 7p included *EGFR* supports that the gene is a likely target of those gains. In the absence of a gain on 7p, cases wild-type for both *EGFR* and *KRAS* either demonstrated amplification of *KRAS* or were characterized by low levels of aberrant genome. The targeting of *EGFR* or *KRAS* appears a nearly constant finding when tumors display genomic instability. However, it has been shown that the molecular subsets defined by *EML4-ALK*, *EGFR*, or *KRAS* mutations are distinct [47].

MCR of gains outnumbered MCR of loss, although the proportions of gained and lost genome were similar, suggesting a greater dispersion of losses. The predominance of gains is observed in most studies [21], [30], [32]. It is likely that other mechanisms inactivate tumor suppressor genes. Copy-neutral LOH may be such a mechanism. Copy-neutral LOH (also known as uniparental disomy)—wherein the retained homolog is duplicated so as to preserve two total copies per cell—is quite common in some cancers [48]. The SNP array analysis revealed recurrent copy-neutral LOH. Among tumor suppressor genes altered by copy-number losses, *RB1* and *WRN* were also present in regions of recurrent copy-neutral LOH. This observation may be meaningful as copy-neutral LOH can be biologically equivalent to the second hit in the Knudson hypothesis. The variety of different genetic events underlying LOH at the *RB1* locus in retinoblastoma seems to occur in lung cancer [49]. On the other hand, at less than 75% tumor DNA in heterogeneous samples an allelic duplication event and an allelic LOH bear resemblance to each other [50]. A comparison between smokers and never smokers with lung carcinoma is required to determine whether LOH is less frequent in never smokers as suggested by the early work of Sanchez-Cespedes et al. [20].

In conclusion, new regions of interest, some of which contained cancer genes or few potential candidate genes, were uncovered. Our results do not establish that the new regions were characteristic of never smoker status, but provide interesting insights into genomic imbalances in lung cancer. Amplicons at 16p11.2 were joined in a HSR including *FUS*, which was over-expressed when the gene was included in 16p11.2 gains. We also showed heterogeneity in lung adenocarcinoma in never smokers with *MYC* as important in the classification. Genetic alterations targeting the *EGFR* signaling pathway appear nearly constant in tumors with genomic instability.

### LG participants, all in France

Centre Chirurgical Marie-Lannelongue, Le Plessis-Robinson: P Dartevelle, E Dulmet, F Leroy-Ladurie, V de Montpreville; CHI Créteil: I Monnet; CHU Dijon,: A Bernard, F Piard; CHU Hôtel-Dieu, Paris: M Alifano, S Camilleri-Broët, JF Régnard; CHU Nice,: P Hofman, V Hofman, J Mouroux; CHU Saint-Louis, Paris: J Trédaniel; CHU Strasbourg,: M Beau-Faller, G Massard, A Neuville; CHU Tenon, Paris: M Antoine, J Cadranel; CHU Toulouse,: L Brouchet, J Mazières, I Rouquette; HIA Percy, Clamart: P Saint-Blancard, F Vaylet; Institut Gustave-Roussy, Villejuif: A Berhneim, P Dessen, F Dufour, N Dorvault, P Fouret, B Job, L Lacroix, V Lazar, C Richon, V Roux, P Saulnier, E Taranchon, S Toujani, A Valent; Institut Mutualiste Montsouris, Paris: P Girard, D Gossot, P Validire; Ligue Nationale contre le Cancer: J Laffaire.

## Supporting Information

Figure S1 **Correlations between percentages of gain and percentages of loss in the whole genome in never smokers with lung adenocarcinoma.** R2: Pearson correlation coefficient. Panel A. Correlation among the 5 clusters A1, A2, B1, B2 and B3. Panel B. Correlation among the 4 clusters A2, B1, B2 and B3 after exclusion of cases with low levels of aberrant genome (<5%) belonging to cluster A1.
(TIF)

Figure S2 **Cluster dendogram with adjusted unbiased (AU) and bootstrap (BP) values (%) in 60 never smokers with lung adenocarcinomas using the R environment package Pvclust.** Distance: euclidean. Cluster method: Ward. BP values (right, green color), AU values (left, red color), and cluster labels (bottom). The AU value may be lower than the BP value when the similarities involve a small proportion of the data. An example is provided by cases 37818 and 37892 belonging to cluster B1, whose region of similarity (8q) was narrow as shown in the heatmap.
(TIF)

Figure S3 **Percentages of aberrant genome in each cluster.** Mean and standard deviation bars. P value: F test.
(TIF)

Figure S4 **Distribution of mutations or gains involving** ***EGFR*** **or** ***KRAS*** **in 57 never smokers with lung adenocarcinoma and available** ***EGFR*** **and** ***KRAS*** **sequencing data.**
(TIF)

Figure S5 **Example of the results of the comparison between aCGH and SNP array profiles in the 7p12.1 region displaying an amplification including** ***DKFZp564N2472*** **.** Lanes from top to bottom: Illumina SNP array profile (log2ratio, slide 35), Agilent aCGH profile (log2ratio, slide 37492), location of Illumina SNP probes, location of Agilent aCGH probes, human genes (plus strand), cytoband and coordinates, human genes (minus strand).
(TIF)

Figure S6 **Box plots of expression levels of 4 Affymetrix probe sets interrogating** ***FUS*** **.** Gain0: no gain of the 16p11.2 region; gain+:gain of the 16p11.2 region. Horizontal line: median; solid circle: mean; upper/lower whiskers: Max/Min value. P values: Student's t test.
(TIF)

Material and Methods S1 **Detailed methods on inclusion of patients, processing of samples,** ***EGFR*** **and** ***KRAS*** **sequencing, oligonucleotide aCGH analysis, genomic PCR, fluorescent in situ hybridization studies, gene expression analysis and SNP array analysis.**
(DOC)

Table S1 (DOC)

Table S2 (TIF)

Table S3 (DOC)

Table S4 (DOC)

Table S5 (DOC)

Table S6 (XLS)
