# Supplementary figures and images for: Genomic Aberrations in Lung Adenocarcinoma in Never Smokers
Source: PLoS One. 2010 Dec 6;5(12):e15145. doi: 10.1371/journal.pone.0015145 (PMC2997777; doi:10.1371/journal.pone.0015145)

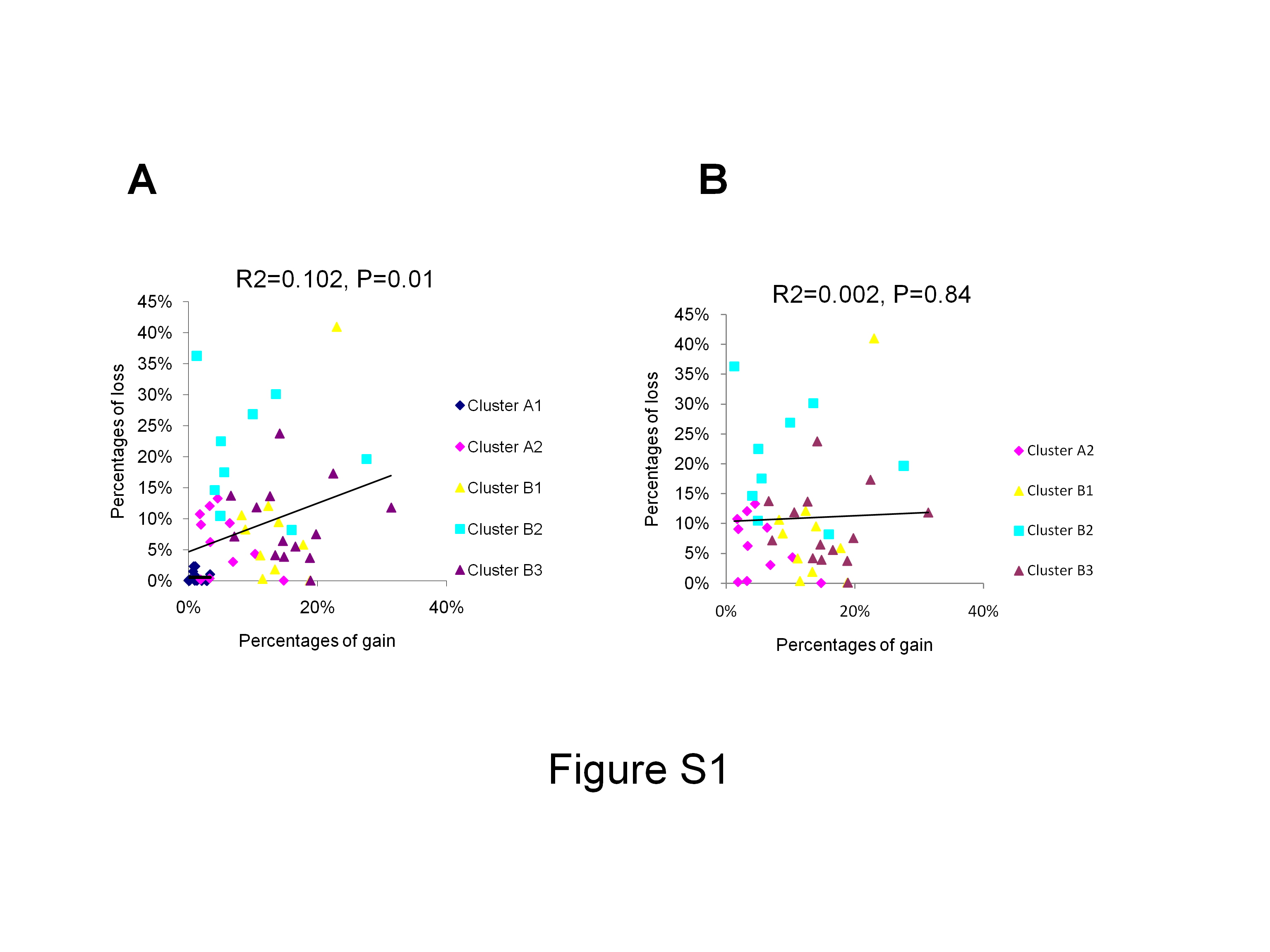

Supplement: Figure S1 — Correlations between percentages of gain and percentages of loss in the whole genome in never smokers with lung adenocarcinoma. R2: Pearson correlation coefficient. Panel A. Correlation among the 5 clusters A1, A2, B1, B2 and B3. Panel B. Correlation among the 4 clusters A2, B1, B2 and B3 after exclusion of cases with low levels of aberrant genome (<5%) belonging to cluster A1. (TIF) [file pone.0015145.s001.tif]

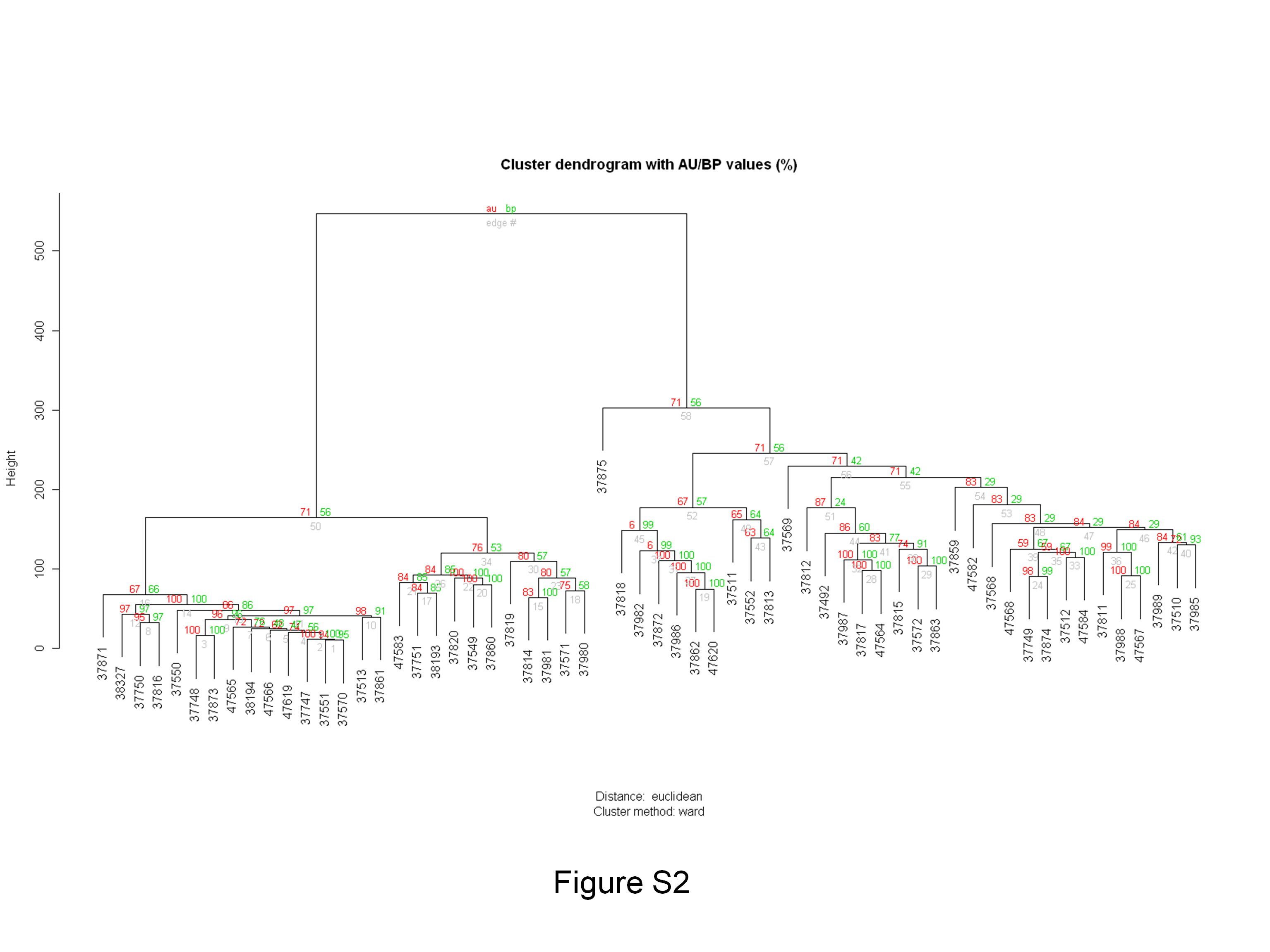

Supplement: Figure S2 — Cluster dendogram with adjusted unbiased (AU) and bootstrap (BP) values (%) in 60 never smokers with lung adenocarcinomas using the R environment package Pvclust. Distance: euclidean. Cluster method: Ward. BP values (right, green color), AU values (left, red color), and cluster labels (bottom). The AU value may be lower than the BP value when the similarities involve a small proportion of the data. An example is provided by cases 37818 and 37892 belonging to cluster B1, whose region of similarity (8q) was narrow as shown in the heatmap. (TIF) [file pone.0015145.s002.tif]

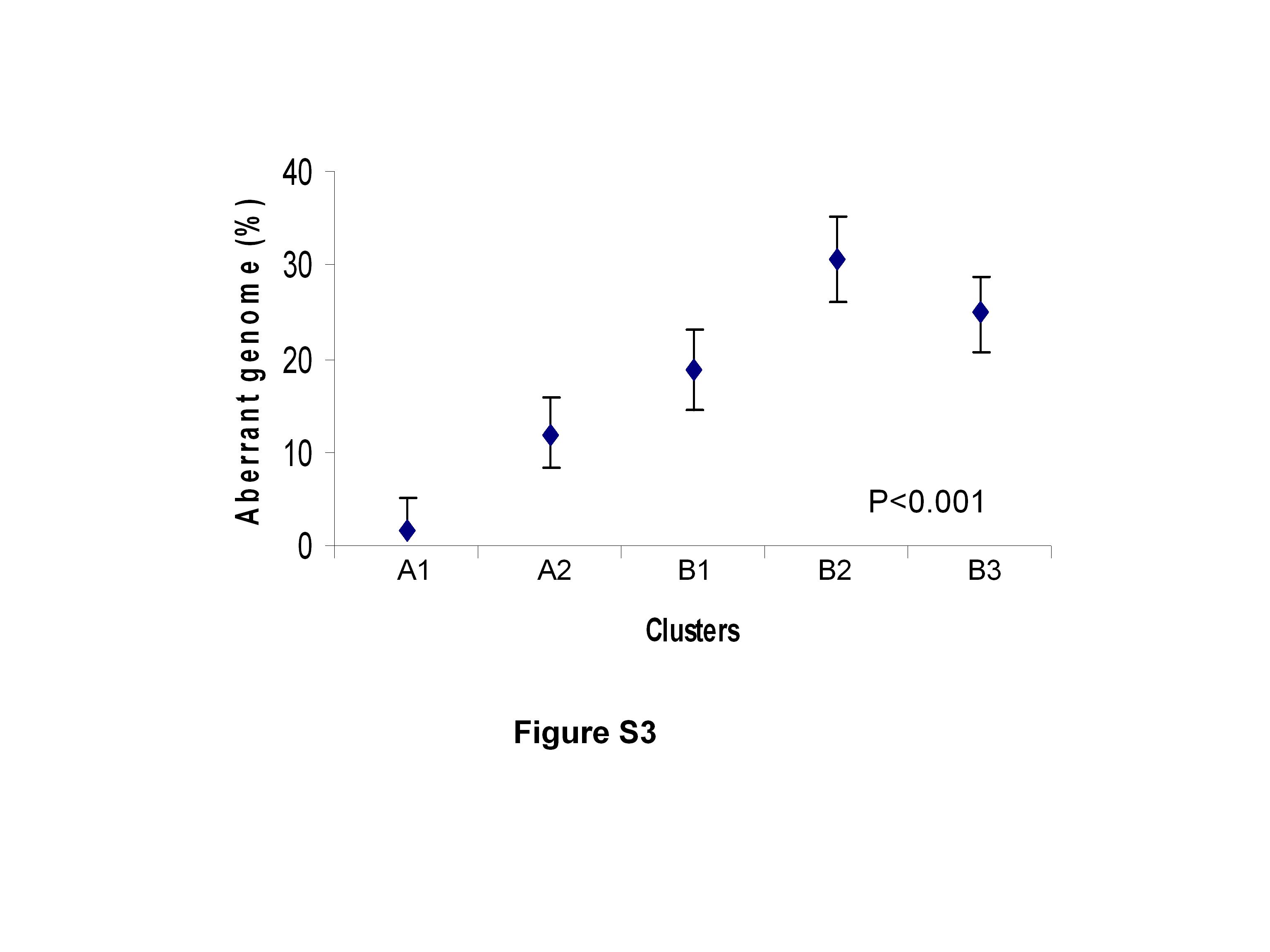

Supplement: Figure S3 — Percentages of aberrant genome in each cluster. Mean and standard deviation bars. P value: F test. (TIF) [file pone.0015145.s003.tif]

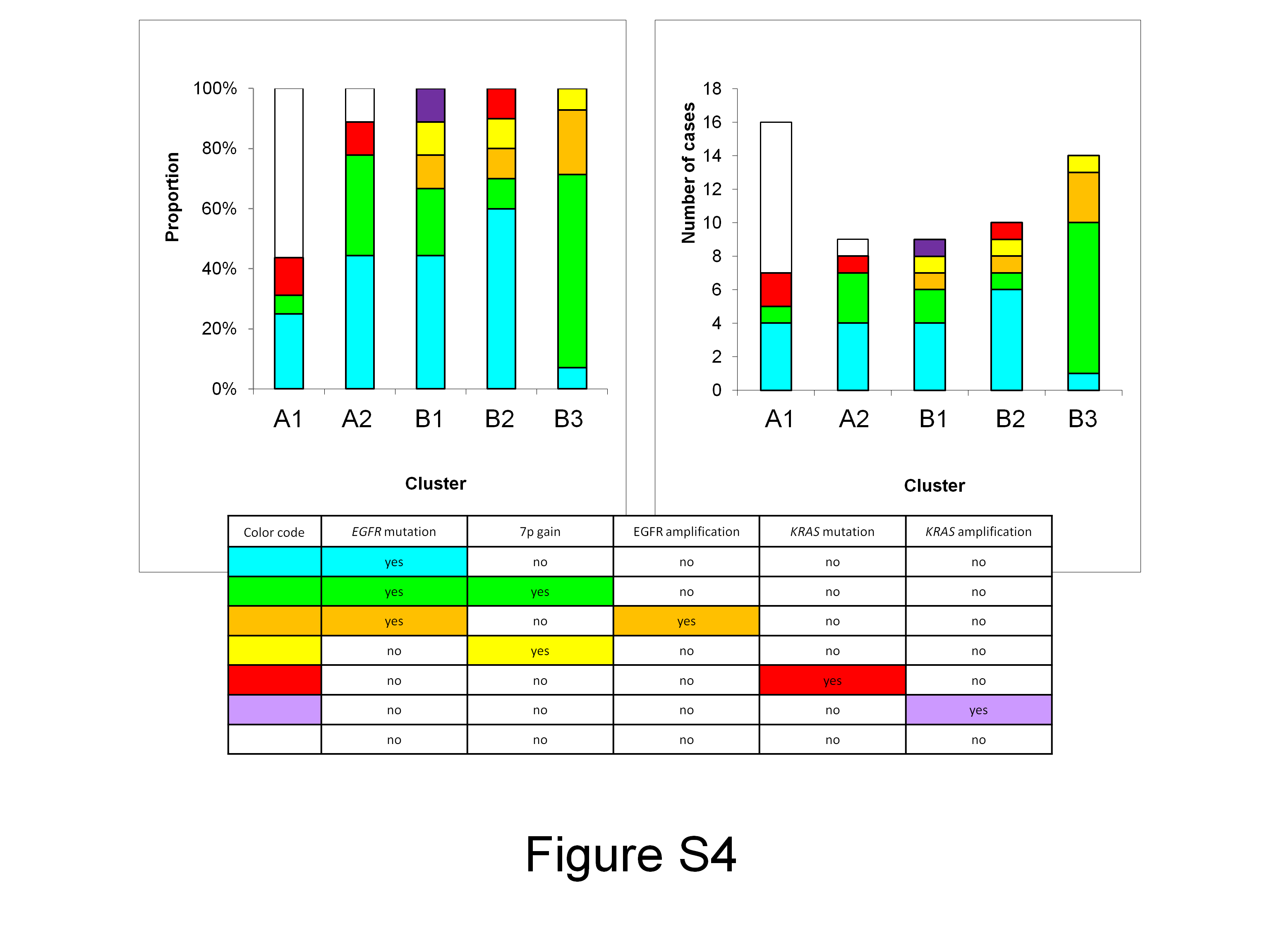

Supplement: Figure S4 — Distribution of mutations or gains involving EGFR or KRAS in 57 never smokers with lung adenocarcinoma and available EGFR and KRAS sequencing data. (TIF) [file pone.0015145.s004.tif]

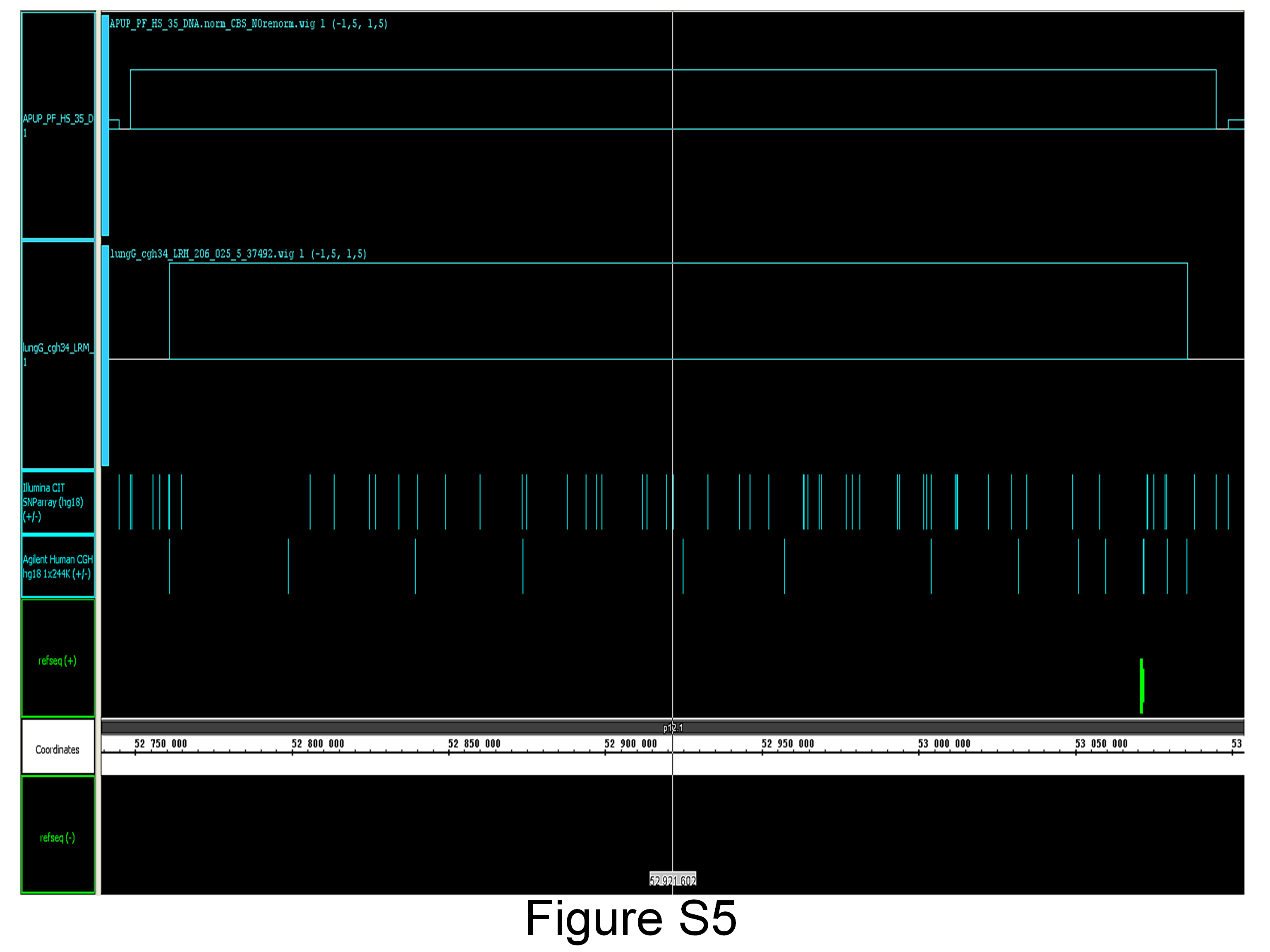

Supplement: Figure S5 — Example of the results of the comparison between aCGH and SNP array profiles in the 7p12.1 region displaying an amplification including DKFZp564N2472 . Lanes from top to bottom: Illumina SNP array profile (log2ratio, slide 35), Agilent aCGH profile (log2ratio, slide 37492), location of Illumina SNP probes, location of Agilent aCGH probes, human genes (plus strand), cytoband and coordinates, human genes (minus strand). (TIF) [file pone.0015145.s005.tif]

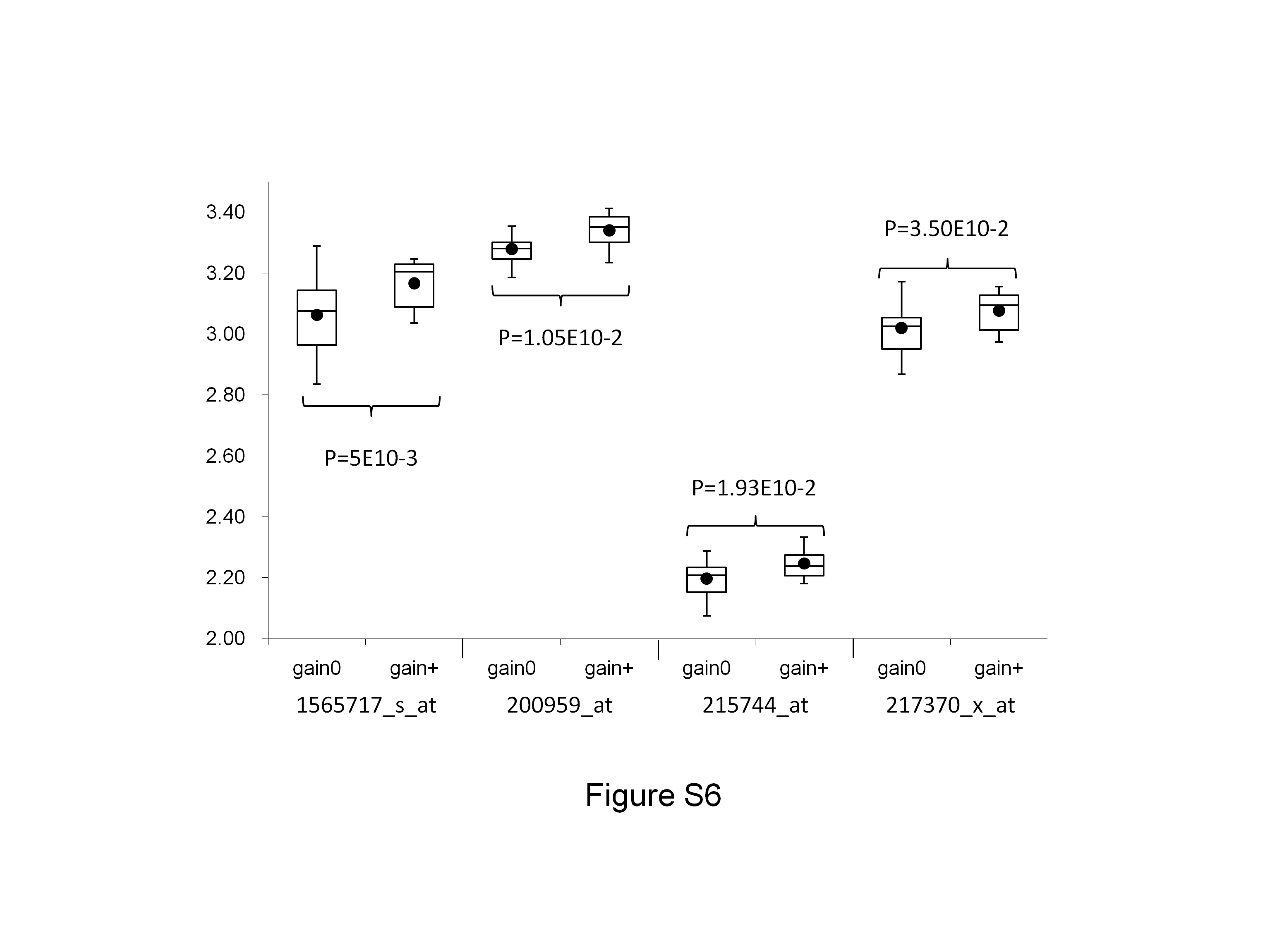

Supplement: Figure S6 — Box plots of expression levels of 4 Affymetrix probe sets interrogating FUS . Gain0: no gain of the 16p11.2 region; gain+:gain of the 16p11.2 region. Horizontal line: median; solid circle: mean; upper/lower whiskers: Max/Min value. P values: Student's t test. (TIF) [file pone.0015145.s006.tif]

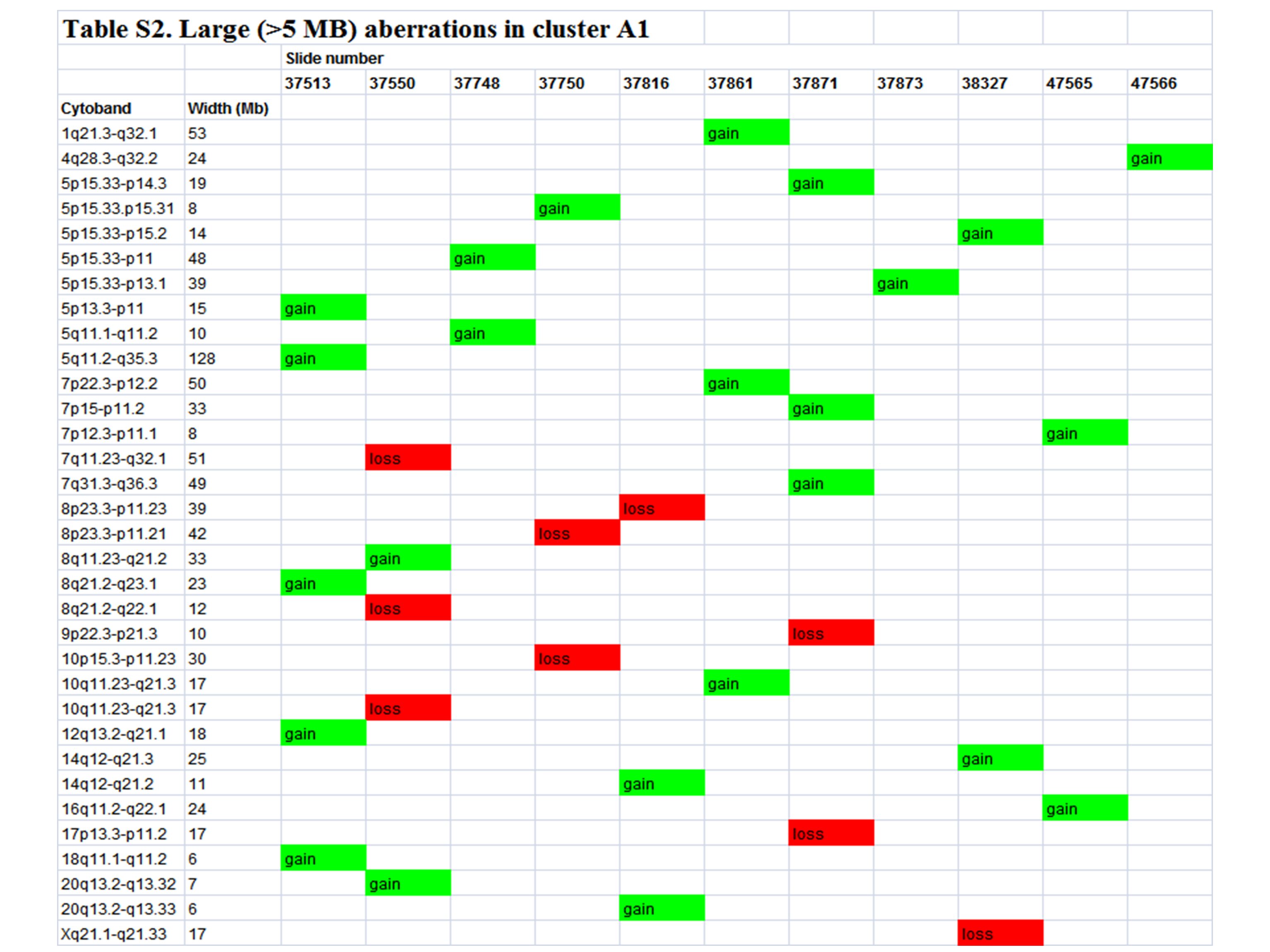

Supplement: Table S2 — (TIF) [file pone.0015145.s009.tif]
