# Supplementary material for: Genomic Aberrations in Lung Adenocarcinoma in Never Smokers
Source: PLoS One. 2010 Dec 6;5(12):e15145. doi: 10.1371/journal.pone.0015145 (PMC2997777; doi:10.1371/journal.pone.0015145)
Supplement: Material and Methods S1 — Detailed methods on inclusion of patients, processing of samples, EGFR and KRAS sequencing, oligonucleotide aCGH analysis, genomic PCR, fluorescent in situ hybridization studies, gene expression analysis and SNP array analysis. (DOC) [file pone.0015145.s007.doc]

**Material and Methods S1**

**Patients**

The project, referred as the Lung Genes study (LG study), was designed to study lung cancer in never smokers as part of a larger program (Programme National d’Excellence Spécialisée Poumon, or PNES Poumon) that was initiated by the Institut National du Cancer (INCa, France). The present collaborative work involved 13 centers in France (a list of participants is shown at the end of the manuscript).

The 60 included patients were never smokers who had a lifetime exposure of less than 100 cigarettes. A first selection of never smokers with surgically removed lung adenocarcinoma was made using the records that were kept at the centers where the patients have been originally treated. Then, smoking status was carefully cross-validated by using a purposely designed simple form that was filled during an interview with the patient or its referring practitioner. Second-hand smoke from the spouse or in the workplace was noted when spontaneously mentioned by the patient.

Cases were included after written informed consent was obtained from the patients following the INCa guidelines and according to French law. All patients had been treated by surgery. Patients who have been previously treated for lung cancer were excluded. No patient had been treated by induction chemotherapy before surgery. Cases with a previous treatment with chemotherapy for another malignancy ending less than one year before the diagnosis of lung cancer or with a history of ipsilateral radiotherapy after mastectomy were also excluded.

The pathological diagnosis was centrally reviewed by an experienced pathologist with the help of histochemical and immunohistochemical stains. Thyroid transcription factor 1 (TTF-1) stain was performed in every case. Staining for thyroglobulin was used to separate metastatic thyroid carcinoma from TTF-1 positive adenocarcinoma of the lung. When TTF-1 stain was negative, a large panel of immunohistochemical markers was used to exclude metastatic adenocarcinomas or epithelioid malignant mesothelioma, and cases for which a doubt about the primary site in the lung remained were excluded.

The main clinical or pathological variables included age, sex, tumor location, surgery type, pathological T, N, M, and stage group according to the TNM classification system [1]. The histological subtypes were noted as well as central scarring and degree of differentiation. A bronchioloalveolar pattern was recorded when present in adenocarcinomas of the mixed histological subtype.

**Samples**

The tumors were sampled by pathologists at each surgical center and were collected at Institut de Cancérologie Gustave-Roussy, where they were processed. Frozen samples at least 3 x 3 x 3 mm in size were cut in a cryostat after removing most of the embedding medium. Beginning and end sections were stained with H&E to assess the proportions of tumor cells. Only cases with an average percentage of tumor cells equal to or above 50% were included. Thirty to sixty sections were placed in two separate tubes and kept frozen in liquid nitrogen. The remaining tissue samples were minimally thawed and touch imprinted onto glass slides, which were air-dried and fixed using a methanol : acetic acid solution.

After homogenization of the frozen sections, genomic DNA and RNA were extracted according to Quiagen protocols. Restriction of DNA, quality control of restricted DNA by microanalysis, pooling, and clean up, were performed following Agilent instructions. Quality control of RNA was performed using Agilent bioanalyzer.

Genomic DNA and RNA were extracted from the HCC827 cell line, obtained from ATCC (Manassas, VA, USA). The cell line was authenticated by comparison of its Agilent aCGH profile with the previously published whole genome tiling path aCGH profile [2].

**Sequencing of *EGFR* and *KRAS***

For *EGFR* and *KRAS*, direct sequencing was performed after PCR amplification of *EGFR* exons 18, 19, 20, 21 (NM_005228.3) and *KRAS* exons 2 and 3 (NM_033360.2), respectively. Purified DNA was sequenced using BigDye® Terminator Cycle Sequencing Kit (Applied Biosystems, Foster City, CA, USA). Sequencing reactions were analyzed on 16-capillary ABI3130 or on 48-capillary 3730 DNA Analyzer® in both sense and antisense directions from at least two independent amplifications. Sequences reading and alignment were performed with SeqScape® software (Applied Biosystems).

## Oligonucleotide aCGH analysis

Genomic DNA was analyzed using 244K Whole Human Genome (G4411B) microarrays (Agilent Technologies, Santa Clara, CA, USA). In all experiments, sex-matched DNA from human female or male individual (Promega, Madison, WI) was used as the reference. Oligonucleotide aCGH processing was performed as detailed in the manufacturer's protocol (version 4.0; [http://www.agilent.com](http://www.agilent.com/)). The hybridized and washed array slide was scanned with an Agilent MicroArray Scanner G2505B (Agilent Technologies, Inc.), then fluorescence signals acquisition and data normalization were performed from the scanned TIFF images using Feature Extraction software (v10.1, protocol CGH_v4_Apr08, Agilent). Raw data text files from the latter were then imported for analysis into CGH Analytics (v3.4.40, Agilent). The data are described in accordance with MIAME guidelines and have been deposited in ArrayExpress (<http://www.ebi.ac.uk/arrayexpress>) under E-TABM-926 accession number.

Aberrations were detected with the ADM-2 algorithm [3] at threshold 6.0 and using feature-level filtering (default parameters) and aberration-level filtering with a minimum of 5 consecutive probes and abs(log2ratio) > 0.25 (all other parameters set to default, except for Centralization and Fuzzy Zero both set to OFF). Amplifications were considered for aberrant regions showing a log2(ratio) > 1.58. Minimal common regions (MCR) were identified with STAC v1.2 [4] and by using both the frequency-confidence and footprint methods at lower and higher stringencies (confidence >0.95 and >0.995, respectively). The results were then combined and manually curated to list every putative MCR. MCR defined by 5 probes or less were excluded. Coincident MCR of gain and loss were discarded as confirmed copy-number variants (CNV). Then, individual cases contributing to the delineation of each MCR of gain or loss were identified. Their profiles were manually reviewed to validate breakpoints and to further discard CNVs, which were recognized by querying the Database of Genomic Variants (<http://projects.tcag.ca/variation/>) [5].

Frequency of aberrations along the genome, hierarchical clustering of samples, and the corresponding heatmap graphical representation were performed using R software (v2.8.0, <http://www.r-project.org/>). For hierarchical clustering, Euclidean distances and Ward’s construction method were used. The bootstrap tests were performed using the R environment package Pvclust [6]. The identification of regions associated with clusters was performed using ANOVA with P values adjusted for their false-discovery rate (FDR) using the Benjamini-Hochberg method[7]. The P values (F-test) for the association of clusters with clinicopathological variables were adjusted for multiple testing using Bonferroni correction.

**Genomic PCR**

Quantification of *FUS* genomic DNA (NC_000016.8) was performed in quadruplicate Taqman® assays using the *AQP8* (NC_000016.8) and *AMPD2* (NT_019273.18) genes as references. The primers and probes were designed using Primer3 software:

- *FUS*, F-CCTACACGCTCTTCCTCCAG

R-TTCCATTTTCCCTGAGATGC

Probe-CCGTTGGAAGCTTCATGTCCTTTCTTC

- *AQP8* F-CCTACACGCTCTTCCTCCAG

R-TTCCATTTTCCCTGAGATGC

Probe-CCTGGGGGCTGGGCTTTGA

- *AMPD2* F-CAGAGGACAGTGGTGAGCAA

R-CTTACTCTTTGGCCAGCCAG

Probe-GGCCAACCTGGGGCTGTGGG

## Fluorescence *in situ* hybridization (FISH) studies

Commercial probes localized around *FUS* (centromeric and telomeric Vysis LSI FUS Dual Color, Break Apart Rearrangement Probe, Abott Laboratories, Abott Park, IL, USA) and two BAC clones RP11-347C12 (GenBank: AC106782) and RP11-388M20 (GenBank: AC009088.9) from 16p11.2 were selected. The position of the commercial probes and the BAC clones are shown in figure 2, panel A. The quality of the probes was verified by hybridization to metaphase spreads and nuclei of a negative control. FISH analysis was carried out on cytological touch preparations. Purified BAC DNA was amplified with Phi29 DNA polymerase by the technique of rolling circle amplification and labeled by random priming in the presence of Alexa 488-dUTP (green) and Alexa 594-dUTP (red) (Abbott). The preparations were observed with an epifluorescence microscope and images captured with a Vysis imaging station. Ten to 20 metaphase spreads and at least 100 nuclei were analyzed for each hybridization.

**Gene expression analysis**

The gene expression analysis encompassed HG-U1133 plus 2.0 Affymetrix array data in a subset of 40 samples belonging to an ongoing study (not published). Affymetrix raw files of the samples were normalized in batch by the use of the Robust Multichip Average method implemented in BrB Array Tools (htpp://linus.nci.nih.gov/BRB-ArrayTools.htlm). Expression of probe sets corresponding to selected genes in the 16p11.2 region were compared with the t-test function implemented in R.

Quantification of *FUS* mRNA expression (NM_004960.2) was performed in duplicate in pre-designed Taqman gene expression assays (Hs01100224_m1, Applera, Villebon-sur-Yvette, France) using *PPIA* mRNA expression as reference.

## SNP array analysis

SNP array genotyping was carried out using the Illumina “HumanCNV370-Quad” array (Illumina, Inc., San Diego, CA) on the Integragen microarray platform (Evry, France) according to the Illumina procedures. Scans were performed on the BeadArray Reader and data were extracted and normalized with Beadstudio software V3 by using standard settings. Data normalization was improved using the normalization procedure tQN proposed by Staaf et al. [8] to make allelic frequencies symmetrical. The circular binary segmentation (CBS) algorithm (DNAcopy R package version 1.20.0 [www.bioconductor.org] was applied to log ratios (LRR) and allelic frequencies (BAF) data. The minimal level of significance was defined as 10^-2^ for LRR and 10^-3^ for BAF. To obtain one banded BAF profile, mirrored BAF was processed and non informative homozygous SNPs were removed as described in Staaf method [9].

Genomic events and copy numbers were recognized using the GAP pattern-recognition algorithm [10]. For assessment of copy-neutral loss of heterozygosity (LOH), only segments with at least 10 consecutive SNPs showing a loss of heterozygosity and a copy number equal to 2 were considered. For readability, the frequencies of SNPs associated with the same locus were averaged.

**References**

1. TNM Atlas (1998) Guide illustré de la classification TNM/pTNM des tumeurs malignes. 4 éd. Paris: Springer-Verlag France.

2. Garnis C, Lockwood WW, Vucic E, Ge Y, Girard L, et al. (2006) High resolution analysis of non-small cell lung cancer cell lines by whole genome tiling path array CGH. Int. J. Cancer 118: 1556-1564.

3. Lipson D, Aumann Y, Ben-Dor A, Linial N, Yakhini Z (2006) Efficient calculation of interval scores for DNA copy number data analysis. J. Comput. Biol 13: 215-228.

4. Diskin SJ, Eck T, Greshock J, Mosse YP, Naylor T, et al. (2006) STAC: A method for testing the significance of DNA copy number aberrations across multiple array-CGH experiments. Genome Res 16: 1149-1158.

5. Iafrate AJ, Feuk L, Rivera MN, Listewnik ML, Donahoe PK, et al. (2004) Detection of large-scale variation in the human genome. Nat. Genet 36: 949-951.

6. Suzuki R, Shimodaira H (2006) Pvclust: an R package for assessing the uncertainty in hierarchical clustering. Bioinformatics 22: 1540-1542.

7. Hochberg Y, Benjamini Y (1990) More powerful procedures for multiple significance testing. Stat Med 9: 811-818.

8. Staaf J, Vallon-Christersson J, Lindgren D, Juliusson G, Rosenquist R, et al. (2008) Normalization of Illumina Infinium whole-genome SNP data improves copy number estimates and allelic intensity ratios. BMC Bioinformatics 9: 409.

9. Staaf J, Lindgren D, Vallon-Christersson J, Isaksson A, Göransson H, et al. (2008) Segmentation-based detection of allelic imbalance and loss-of-heterozygosity in cancer cells using whole genome SNP arrays. Genome Biol 9: R136.

10. Popova T, Manié E, Stoppa-Lyonnet D, Rigaill G, Barillot E, et al. (2009) Genome Alteration Print (GAP): a tool to visualize and mine complex cancer genomic profiles obtained by SNP arrays. Genome Biol 10: R128.
