# Supplementary material for: Genomic Aberrations in Lung Adenocarcinoma in Never Smokers
Source: PLoS One. 2010 Dec 6;5(12):e15145. doi: 10.1371/journal.pone.0015145 (PMC2997777; doi:10.1371/journal.pone.0015145)
Supplement: Table S1 — (DOC) [file pone.0015145.s008.doc]

Table S1. Summary of the main clinicopathological characteristics

| **Age (years)** | |  | **n** |
| --- | --- | --- | --- |
|  | 21-30 |  | 1 |
|  | 31-40 |  | 0 |
|  | 41-50 |  | 2 |
|  | 51-60 |  | 14 |
|  | 61-70 |  | 15 |
|  | 71-80 |  | 21 |
|  | 81-90 |  | 7 |
| **Sex** |  |  |  |
|  | Female |  | 53 |
|  | Male |  | 7 |
| **Localization** | |  |  |
|  | Left lung | |  |
|  |  | Inferior lobe | 17 |
|  |  | Superior lobe | 19 |
|  |  | na | 1 |
|  | Right lung | |  |
|  |  | Inferior lobe | 8 |
|  |  | Superior lobe | 12 |
|  |  | Middle lobe | 2 |
|  |  | Inferior and superior lobes | 1 |
| **Surgery type** | |  |  |
|  | Pneumectomy | | 3 |
|  | Lobectomy | | 53 |
|  | Bilobectomy | | 1 |
|  | Segmentectomy | | 1 |
|  | Wedge resection | | 1 |
|  | Pleurectomy | | 1 |
| **Size (mm)** | |  |  |
|  | 1-10 |  | 2 |
|  | 11-20 |  | 7 |
|  | 21-30 |  | 20 |
|  | 31-40 |  | 16 |
|  | 41-50 |  | 7 |
|  | 51-60 |  | 4 |
|  | 61-70 |  | 1 |
|  | 71-80 |  | 0 |
|  | >80 |  | 2 |
|  | na |  | 1 |
| **TNM*** |  |  |  |
|  | IA |  | 13 |
|  | IB |  | 19 |
|  | IIB |  | 6 |
|  | IIIA |  | 18 |
|  | IIIB |  | 3 |
|  | IV |  | 1 |
| **T** |  |  |  |
|  | T1 |  | 16 |
|  | T2 |  | 37 |
|  | T3 |  | 4 |
|  | T4 |  | 3 |
| **N** |  |  |  |
|  | N0 |  | 33 |
|  | N1 |  | 5 |
|  | N2 |  | 19 |
|  | Nx |  | 3 |
| **M** |  |  |  |
|  | M0 |  | 59 |
|  | M1 |  | 1 |
| **Histological subtype** | |  |  |
|  | Acinar |  | 9 |
|  | Mixed |  | 47 |
|  | Papillary |  | 2 |
|  | Solid |  | 2 |
| **Differenciation** | |  |  |
|  | High |  | 34 |
|  | Intermediate |  | 7 |
|  | Low |  | 19 |
| **Fibrosis** |  |  |  |
|  | Presence |  | 19 |
|  | Absence |  | 40 |
|  | na |  | 1 |
| **NKX2-1 immunohistochemical expression** | | |  |
|  | Present |  | 57 |
|  | Absent |  | 3 |
| ***EGFR*** |  |  |  |
|  | Mutated |  | 40 |
|  | Wild-type |  | 19 |
|  | na |  | 1 |

*6th edition TNM staging system for lung cancer
