# Supplementary material for: Genomic Aberrations in Lung Adenocarcinoma in Never Smokers
Source: PLoS One. 2010 Dec 6;5(12):e15145. doi: 10.1371/journal.pone.0015145 (PMC2997777; doi:10.1371/journal.pone.0015145)
Supplement: Table S3 — (DOC) [file pone.0015145.s010.doc]

**Table S3. Regions containing cancer genes and differentially altered between clusters**

| **Cytoband** | **Start** | **End** | **Width (Kb)** | **Coding genes (n)** | **Cancer genes*** | **Cluster A1** | | **Cluster A2** | | **Cluster B1** | | **Cluster B2** | | **Cluster B3** | | **Adjusted P value^§^** |
| --- | --- | --- | --- | --- | --- | --- | --- | --- | --- | --- | --- | --- | --- | --- | --- | --- |
|  |  |  |  |  |  | **Gain** | **Loss** | **Gain** | **Loss** | **Gain** | **Loss** | **Gain** | **Loss** | **Gain** | **Loss** |  |
| 7p22.2 | 2 855 854 | 3 122 723 | 266 | 1 | CARD11 | 6% | 0% | 27% | 0% | 50% | 0% | 33% | 0% | 78% | 0% | 5.89E-03 |
| 7p21.2 | 13 340 912 | 14 155 039 | 814 | 2 | ETV1 | 6% | 0% | 18% | 0% | 50% | 0% | 22% | 0% | 92% | 0% | 1.87E-05 |
| 7p12.2 | 50 310 128 | 50 461 515 | 151 | 1 | IKZF1 | 18% | 0% | 27% | 0% | 50% | 0% | 33% | 0% | 85% | 0% | 4.77E-03 |
| 7q11.23 | 72 388 059 | 75 477 666 | 3090 | 50 | ELN, HIP1 | 0% | 0% | 9% | 9% | 20% | 0% | 22% | 0% | 64% | 0% | 1.38E-03 |
| 7q33 | 136 742 893 | 138 614 323 | 1870 | 14 | CREB3L2 KIAA1549 | 6% | 0% | 0% | 18% | 20% | 10% | 11% | 0% | 64% | 0% | 1.30E-03 |
| 7q34 | 139 844 597 | 141 116 030 | 1270 | 14 | BRAF | 6% | 0% | 0% | 18% | 20% | 10% | 11% | 0% | 64% | 0% | 1.30E-03 |
| 8p12 | 31 041 658 | 31 193 096 | 151 | 1 | WRN | 0% | 12% | 0% | 9% | 20% | 40% | 0% | 88% | 0% | 57% | 1.77E-03 |
| 8q22.2 | 100 898 756 | 101 605 693 | 707 | 7 | COX6C | 12% | 0% | 18% | 0% | 90% | 0% | 33% | 11% | 28% | 7% | 7.83E-04 |
| 8q24.21 | 128 642 695 | 128 838 701 | 196 | 2 | MYC | 6% | 0% | 18% | 0% | 100% | 0% | 33% | 11% | 35% | 0% | 6.00E-05 |
| 9p21.3 | 21 957 748 | 21 957 841 | 0.094 | 1 | CDKN2A | 0% | 25% | 0% | 72% | 10% | 10% | 0% | 66% | 0% | 78% | 3.86E-03 |
| 13q12.2 | 26 746 221 | 29 971 891 | 3230 | 24 | CDX2 | 0% | 0% | 9% | 9% | 0% | 10% | 0% | 77% | 0% | 35% | 2.90E-04 |
| 13q13.1 | 31 589 342 | 32 536 757 | 947 | 9 | BRCA2 | 0% | 6% | 9% | 9% | 0% | 20% | 0% | 88% | 0% | 35% | 2.96E-04 |
| 13q14.2 | 46 994 847 | 49 026 553 | 2030 | 16 | RB1 | 0% | 0% | 9% | 9% | 0% | 10% | 0% | 88% | 0% | 28% | 3.00E-05 |
| 13q33.1 | 102 077 928 | 102 311 685 | 233 | 8 | ERCC5 | 0% | 0% | 9% | 0% | 30% | 0% | 0% | 77% | 7% | 14% | 4.78E-05 |

* Cancer gene census april 2010; § ANOVA with adjustment for false discovery rate
