# Supplementary material for: Genomic Aberrations in Lung Adenocarcinoma in Never Smokers
Source: PLoS One. 2010 Dec 6;5(12):e15145. doi: 10.1371/journal.pone.0015145 (PMC2997777; doi:10.1371/journal.pone.0015145)
Supplement: Table S4 — (DOC) [file pone.0015145.s011.doc]

**Table S4. Mutations in *EGFR* an *KRAS* in 60 never smokers with lung adenocarcinoma^§^**

|  | ***EGFR*** | | | | | | | | | | | | | | | ***KRAS*** | | | | | | |
| --- | --- | --- | --- | --- | --- | --- | --- | --- | --- | --- | --- | --- | --- | --- | --- | --- | --- | --- | --- | --- | --- | --- |
|  | **Exon 18** | | | | | **Exon 19** | | **Exon 20** | | | | | **Exon 21** | | | **Exon 2** | | | | **Exon 3** | | |
|  | CDS Mutation | | | AA Mutation | | CDS Mutation | AA Mutation | CDS Mutation | | AA Mutation | | | CDS Mutation | | AA Mutation | CDS Mutation | | AA Mutation | | CDS Mutation | | AA Mutation |
|  |  | | |  | |  |  |  | |  | | |  | |  |  | |  | |  | |  |
| **N°** | |  |  | |  | |  | |  | |  |  | |  | | |  | |  |  |  | |
| **1** | | WT | WT | | c.2236_2250del | | p.Glu746_Ala750del | | WT | | WT | WT | | WT | | | WT | | WT | WT | WT | |
| **2** | | WT | WT | | c.2237_2255del insT | | p.Glu746_Ser752 del insPhe | | WT | | WT | WT | | WT | | | WT | | WT | WT | WT | |
| **4** | | WT | WT | | c.2237_2255del insT | | p.Glu746_Ser752 insVal | | WT | | WT | WT | | WT | | | WT | | WT | WT | WT | |
| **5** | | WT | WT | | WT | | WT | | WT | | WT | c.2573T>K | | p.Leu858Arg | | | WT | | WT | WT | WT | |
| **7** | | WT | WT | | c.2240_2257del | | p.Leu747_Pro753del insSer | | WT | | WT | WT | | WT | | | WT | | WT | WT | WT | |
| **10** | | WT | WT | | c.2235_2249del | | p.Glu746_Ala750del | | WT | | WT | WT | | WT | | | WT | | WT | WT | WT | |
| **11** | | WT | WT | | c.2236_2250del | | p.Glu746_Ala750del | | WT | | WT | WT | | WT | | | WT | | WT | WT | WT | |
| **12** | | WT | WT | | WT | | WT | | WT | | WT | WT | | WT | | | c.34G>K | | p.Gly12Cys | WT | WT | |
| **13** | | WT | WT | | WT | | WT | | c.2318A>R | | p.His773Arg | WT | | WT | | | WT | | WT | WT | WT | |
| **14** | | WT | WT | | c.2235_2249del | | p.Glu746_Ala750del | | WT | | WT | WT | | WT | | | WT | | WT | WT | WT | |
| **15** | | WT | WT | | c.2236_2250del | | p.Glu746_Ala750del | | WT | | WT | WT | | WT | | | WT | | WT | WT | WT | |
| **16** | | c.2065G>K | p.Val689Leu | | WT | | WT | | WT | | WT | c.2573T>K | | p.Leu858Arg | | | WT | | WT | WT | WT | |
| **18** | | c.2126A>W | p.Glu709Val | | WT | | WT | | WT | | WT | c.2573T>K | | p.Leu858Arg | | | WT | | WT | WT | WT | |
| **22** | | WT | WT | | c.2235_2249del | | p.Glu746_Ala750del | | WT | | WT | WT | | WT | | | WT | | WT | WT | WT | |
| **23** | | WT | WT | | WT | | WT | | c.2311insGGGGAC | | p.Asp770_  Asn771 | WT | | WT | | | WT | | WT | WT | WT | |
| **24** | | WT | WT | | WT | | WT | | WT | | WT | c.2582T>W | | p.Leu861Gln | | | WT | | WT | WT | WT | |
| **27** | | c.2126_  2128del | p.Glu709_Thr710delinsAla | | WT | | WT | | WT | | WT | WT | | WT | | | WT | | WT | WT | WT | |
| **30** | | WT | WT | | c.2235_2249del | | p.Glu746_Ala750del | | WT | | WT | WT | | WT | | | WT | | WT | WT | WT | |
| **32** | | WT | WT | | c.2235_2255del insGGT | | p.Glu746_Ser752 delinsVal | | WT | | WT | WT | | WT | | | WT | | WT | WT | WT | |
| **35** | | WT | WT | | c.2219_2220insTCCCGTCGCTATTAAAAT | | p.Ileu740_Pro741 ins | | WT | | WT | WT | | WT | | | WT | | WT | WT | WT | |
| **39** | | WT | WT | | WT | | WT | | WT | | WT | c.2573T>K | | p.Leu858Arg | | | WT | | WT | WT | WT | |
| **41** | | WT | WT | | c.2236_2250del | | p.Glu746_Ala750del | | WT | | WT | WT | | WT | | | WT | | WT | WT | WT | |
| **44** | | WT | WT | | WT | | WT | | WT | | WT | c.2573T>K | | p.Leu858Arg | | | WT | | WT | WT | WT | |
| **46** | | WT | WT | | c.2236_2248del insCAAC | | p.Glu746_Aladel insGlnPro | | WT | | WT | WT | | WT | | | WT | | WT | WT | WT | |
| **47** | | WT | WT | | c.2236_2250del | | p.Glu746_Ala750del | | WT | | WT | WT | | WT | | | WT | | WT | WT | WT | |
| **48** | | WT | WT | | WT | | WT | | WT | | WT | WT | | WT | | | c.35G>K | | p.Gly12Val | WT | WT | |
| **49** | | WT | WT | | c.2236_2250del | | p.Glu746_Ala750del | | WT | | WT | WT | | WT | | | WT | | WT | WT | WT | |
| **50** | | WT | WT | | WT | | WT | | WT | | WT | WT | | WT | | | WT | | WT | c.182A>W | p.Gln61Leu | |
| **54** | | WT | WT | | WT | | WT | | WT | | WT | c.2573T>K | | p.Leu858Arg | | | WT | | WT | WT | WT | |
| **55** | | WT | WT | | WT | | WT | | WT | | WT | c.2573T>K | | p.Leu858Arg | | | WT | | WT | WT | WT | |
| **56** | | WT | WT | | WT | | WT | | WT | | WT | c.2573T>G | | p.Leu858Arg | | | WT | | WT | WT | WT | |
| **57** | | c.2156G>S | p.Gly719Ala | | WT | | WT | | WT | | WT | c.2582T>W | | p.Leu861Glu | | | WT | | WT | WT | WT | |
| **59** | | WT | WT | | c.2230_2249del insGTCAA | | p.Ileu744_Ala750delinsValLys | | WT | | WT | WT | | WT | | | WT | | WT | WT | WT | |
| **60** | | WT | WT | | c.2235_2249del | | p.Glu746_Ala750del | | WT | | WT | WT | | WT | | | WT | | WT | WT | WT | |
| **61** | | WT | WT | | WT | | WT | | WT | | WT | c.2573T>K | | p.Leu858Arg | | | WT | | WT | WT | WT | |
| **63** | | WT | WT | | c.2239_2265del insGCCAA | | p.Leu747_Lys754 delinsGln | | WT | | WT | WT | | WT | | | WT | | WT | WT | WT | |
| **64** | | WT | WT | | WT | | WT | | WT | | WT | WT | | WT | | | c.35G>S | | p.Gly12Ala | WT | WT | |
| **65** | | WT | WT | | WT | | WT | | WT | | WT | c.2573T>K | | p.Leu858Arg | | | WT | | WT | WT | WT | |
| **68** | | na | na | | na | | na | | na | | na | na | | na | | | WT | | WT | WT | WT | |
| **84** | | WT | WT | | c.2235_2249del | | p.Glu746_Ala750del | | WT | | WT | WT | | WT | | | WT | | WT | WT | WT | |
| **85** | | WT | WT | | WT | | WT | | WT | | WT | c.2582T>A | | p.Leu861Gln | | | WT | | WT | WT | WT | |
| **88** | | WT | WT | | WT | | WT | | c.2356G>A | | p.Val786Met | c,2573T>G | | p.Leu858Arg | | | WT | | WT | WT | WT | |
| **90** | | WT | WT | | WT | | WT | | c,2369C>T | | p.Thr790Met | c,2573T>G | | p.Leu858Arg | | | WT | | WT | WT | WT | |
| **93** | | WT | WT | | c.2240_2257del | | p.Leu747_Pro753delinsSer | | WT | | WT | WT | | WT | | | WT | | WT | WT | WT | |
| **94** | | WT | WT | | c.2236_2250del | | p.Glu746_Ala750del | | WT | | WT | WT | | WT | | | WT | | WT | WT | WT | |

$ Sequencing data not available in 2 cases for *KRAS* and 1 case for *EGFR*
