# Supplementary material for: Genomic Aberrations in Lung Adenocarcinoma in Never Smokers
Source: PLoS One. 2010 Dec 6;5(12):e15145. doi: 10.1371/journal.pone.0015145 (PMC2997777; doi:10.1371/journal.pone.0015145)
Supplement: Table S5 — (DOC) [file pone.0015145.s012.doc]

Table S5. Large regions of gain or loss in previously published studies and in present study

1 : Testa et al. 1994, Balsara et al. 1997, Petersen et al 1997, Björkqvist et al. 1998, Luk et al. 2001, Pei et al. 2001 ; 2 : italics: event reported in more than one CGH study; 3 : underlined: event not found in present study; 4 : * non significant association with smoking status; 5 : §: event frequency >20%; 6 : bold characters: event not reported in the cited studies; 7 : 82 never smokers among 528 cases
